# Tethered and Untethered 3D Microactuators Fabricated by Two-Photon Polymerization: A Review

**DOI:** 10.3390/mi12040465

**Published:** 2021-04-20

**Authors:** Zhaoxin Lao, Neng Xia, Shijie Wang, Tiantian Xu, Xinyu Wu, Li Zhang

**Affiliations:** 1 Department of Mechanical and Automation Engineering, The Chinese University of Hong Kong, Sha Tin, Hong Kong 999077, China; 1155135729@link.cuhk.edu.hk (N.X.); shijiewang1994@163.com (S.W.); 2 Anhui Province Key Laboratory of Measuring Theory and Precision Instrument, School of Instrument Science and Opto-Electronics Engineering, Hefei University of Technology, Hefei 230009, China; 3 CAS Key Laboratory of Mechanical Behavior and Design of Materials, Key Laboratory of Precision Scientific Instrumentation of Anhui Higher Education Institutes, Department of Precision Machinery and Precision Instrumentation, University of Science and Technology of China, Hefei 230022, China; 4 Guangdong Provincial Key Laboratory of Robotics and Intelligent System, Shenzhen Institutes of Advanced Technology, Chinese Academy of Sciences, Shenzhen 518055, China; tt.xu@siat.ac.cn (T.X.); xy.wu@siat.ac.cn (X.W.)

**Keywords:** microactuators, two-photon polymerization, 3D microrobotic

## Abstract

Microactuators, which can transform external stimuli into mechanical motion at microscale, have attracted extensive attention because they can be used to construct microelectromechanical systems (MEMS) and/or microrobots, resulting in extensive applications in a large number of fields such as noninvasive surgery, targeted delivery, and biomedical machines. In contrast to classical 2D MEMS devices, 3D microactuators provide a new platform for the research of stimuli-responsive functional devices. However, traditional planar processing techniques based on photolithography are inadequate in the construction of 3D microstructures. To solve this issue, researchers have proposed many strategies, among which 3D laser printing is becoming a prospective technique to create smart devices at the microscale because of its versatility, adjustability, and flexibility. Here, we review the recent progress in stimulus-responsive 3D microactuators fabricated with 3D laser printing depending on different stimuli. Then, an outlook of the design, fabrication, control, and applications of 3D laser-printed microactuators is propounded with the goal of providing a reference for related research.

## 1. Introduction

Organisms in nature have experienced survival competition for hundreds of millions of years and have evolved their own unique skills for survival, which can sense and respond to changes in the external environment. Inspired by living organisms, people have created a variety of tools that can transform external stimuli into mechanical motion [1]. In recent decades, the actual demand for applications, from in vivo tasks such as minimally invasive surgery and targeted gene delivery to in vitro tasks such as photocatalytic water purification and transporting micro objects, has led to the miniaturization of stimulus-response machines [2]. Therefore, microactuators, which can transform external stimuli into mechanical motion at microscale, have attracted extensive attention due to their attractive potential in a wide variety of fields including bio/chemical sensing, cargo delivery, precision medicine, and biomimetic microrobots.

Thus far, many useful dynamic devices have been created, including smart actuators with moisture-sensitive graphene paper, light-controlled graphene–elastin composite hydrogel actuators, magnetically driven swimmers, and pH-sensitive hydrogel sensors [3,4]. Generally, the construction of microactuators can be classified into two categories: (1) deformable microactuators that typically possess coupled components with different physical/chemical parameters (including expansion rate and Young’s modulus), for example, microgrippers made with two different materials [5,6], and (2) integral moveable microactuators that can move under external stimuli (such as light or a chemical solution), including helix swimmers that can move under the control of magnetic fields [7,8]. Microactuators can be manipulated by various external stimuli such as magnetic fields, light, humidity, pH values, chemicals, thermal conditions, electric fields, and the combination of two or more of these stimuli.

Although many techniques have been applied to the preparation of miniaturized moving machines, [9] the process still faces many challenges when the size of the microactuator is reduced to the level of microns (from 1 μm to 1 mm) [10]. Two-dimensional photolithography and its derivative techniques (such as UV lithography [6]) have been used to manufacture 2D microactuators with a layer-by-layer procedure [11]. For example, Ma et al. reported a moisture-responsive graphene actuator swarm that could achieve complex and predictable deformation by UV lithography [12,13]. Combined with origami crafting, 2D photolithography has been also employed to fabricate 3D microactuators with a self-assembly process [5,11,12]. Although the aforementioned actuators can deform into 3D structures on the basis of crosslink gradients, they are essentially 2D devices. These inherent issues significantly limit their applications in 3D microrobots and energetic 3D microactuators. Permitting the designable prototyping of arbitrary 3D microstructures will render these microactuators smarter.

In recent years, some 3D printing methods have been proposed for the construction of 3D microstructures (Table 1) [14,15,16,17]. Smart materials are especially suitable materials for microactuators because they can respond directly to external stimuli [18,19,20,21]. Merging these two fields has given rise to a new strategy to fabricate microactuators: 4D printing [14,22,23]. However, current reported 4D printing techniques are mostly used to prepare structures with sizes in the range from tens of micrometers to several millimeters, and microactuators with sub-micron features, which are crucial for a fast response speed, are difficult to fabricate. Another challenge of this type of 4D printing is the mismatching of the geometry and the inner stress in printed structures because of multi-material and/or layer-to-layer printing processing. More importantly, the crosslinking densities of material in 4D ink printing are narrowly balanced. If the crosslinking densities are too low, the printed structure will be too soft to have 3D architectures. If the crosslinking densities are too high, the material will be very difficult to extrude from the print needle and it will be difficult to use the material in the construction of freestanding 3D shapes with excellent deformability [24]. Therefore, a facile 4D printing strategy that features complex 3D geometry, microscale dimension, high flexibility, and versatility in terms of materials should be studied.

Femtosecond laser (fs-laser) two-photon polymerization (TPP) is based on the two-photon absorption (TPA) of materials. Polymerization occurs only in the vicinity of the focal spot, and the size of solidified voxels (three-dimensional volume elements) is reduced because of the quadratic dependence of TPA probability on the photon fluence density. By using nonlinear processes of the photochemical reactions involved, the resolution of TPP can exceed the diffraction limit of Rayleigh criterion [25]. Therefore, fs-laser TPP features nanometer spatial resolution, an ultralow thermal effect, and excellent geometry designability [25,26,27]. Three-dimensional fs-laser printing is an attractive technique for the precise fabrication of complex structures at microscale and nanoscale with high resolution [28,29,30,31,32,33,34,35,36,37]. By employing fs-laser printing, tremendous efforts have been made to create 3D microstructures for versatile applications. For a stimulus-responsive structure, the designed shape and the structural inhomogeneity jointly dominate the deformation characteristics of the structure [38,39]. Compared with other fabrication strategies, a significant advantage of fs-laser printing is that the laser parameters, including the light dose, exposure time, scanning path, and distance, can be varied instantaneously so that true 3D microstructures can be realized easily [24,40].

The performance of an actuator is dominated by the structural heterogeneity and the 3D shape, which can be tailored flexibly with the laser exposure dose and the scanning path, respectively. All these functions render 3D laser printing a promising tool for fabricating 3D microactuators. Two-photon polymerization (TPP) permits the versatile maskless 3D printing of microscale structures with features as small as 100 nm [25,26]. A number of reports have been published demonstrating the use of this technique to fabricate a wide range of different microrobots.

Considering the above advantages, 3D laser printing is considered as a very promising technology for the manufacturing of 3D microactuators, and a large amount of interesting work has been done [41]. Although many inspiring reviews about 3D microrobotics have been made by researchers [42,43,44,45,46,47,48,49], few articles focused on 3D microactuators made with 3D laser printing based on TPP.

The employment of 3D laser printing for 3D microactuators not only provides a flexible and powerful tool to fabricate new stimulus-responsive devices, but also paves a new path for the design of the operation mechanisms of 3D microactuators. In this review, we discuss the recent progress of stimulus-responsive 3D microactuators fabricated with 3D laser printing depending on different stimuli in Section 2. Then, the outlook of the design, fabrication, control, and application of 3D laser-printed microactuators is propounded in terms of the future focus in Section 3. Lastly, in Section 4, we conclude with current challenges and perspectives about 3D laser-printed microactuators.

## 2. Stimulus Methods of Microactuators

Internal-combustion engines driven by chemical energy and electric motors driven by electricity have achieved great success in the driving of macroscopic objects, but when the size of objects is reduced to the micro–nano size, the actuation of microactuators becomes complicated and difficult in environments with a low Reynolds number (*Re*). The *Re* is a dimensionless number used to represent the flow of a fluid (gas or liquid) in which an object is moving. The *Re* can be calculated as

(1) $$Re=\frac{\rho vL}{\mu },$$

where ρ and μ refer to the density and viscosity of the fluid, respectively, while v and are the speed and the characteristic length of the moving object in the fluid. The *Re* can be viewed as the ratio of the inertial force to the viscous force when an object is moving in a fluid. The larger the *Re* value is, the less viscous force the object is subject to and the easier the object is to drive. In contrast, the smaller the *Re* is, the more viscous resistance the object is subject to in its motion, resulting in the need to continuously consume energy to maintain its motion. However, for a micro or nano object, it is a challenge to attach a power source to the object. Therefore, one of the most reasonable strategies for micro/nano actuators to acquire enough locomotion power is to harvest energy from the surrounding environment.

**Figure 1:**
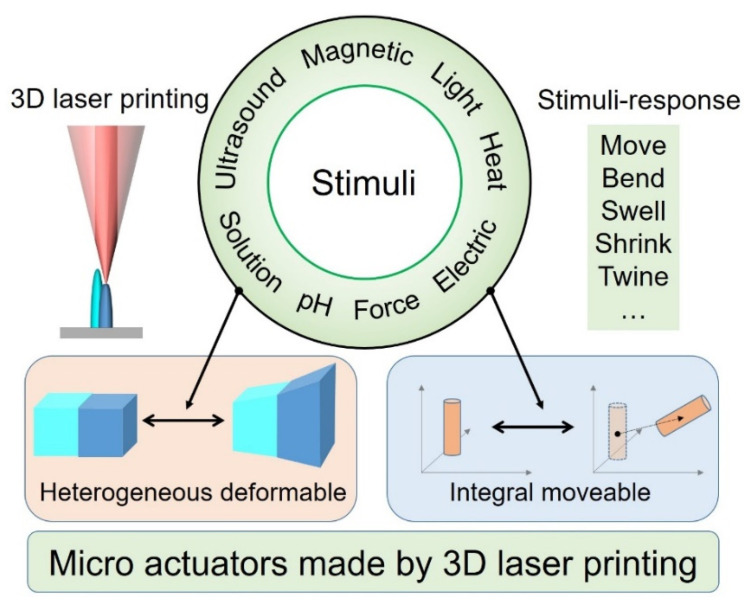
The construction and stimulus method of microactuators made by 3D laser printing.

Here, we review the recent progress of 3D microactuators fabricated with TPP and classify them according to the mode of replenishing energy from the environment, including magnetic, light, acoustic, electric, heat, PH, solution, and microforce (Figure 1).

### 2.1. Magnetic Field

Due to the geomagnetic field, organisms are so accustomed to the magnetic field that they are always unaware of it, which means that magnetic actuation is transparent, external, remotely controllable, adaptive to motion, and, most importantly, life-friendly. The abovementioned advantages make magnetic actuation the preferred strategy for driving microactuators [58]. A large amount of exploratory work has been done and continues to be carried forward [42,59,60,61,62,63,64,65,66,67,68]. The marriage of the magnetic driving method and TPP, which features precise and designable 3D shapes for microstructures, provides a new platform for the research of magnetron microactuators.

**Figure 2:**
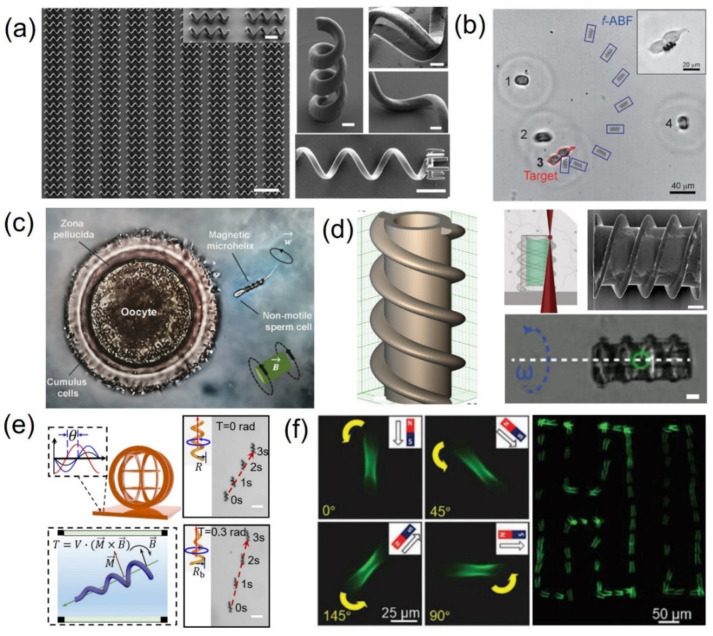
(**a**) Array and locally amplified microhelical micromachines printed by TPP. Magnetic material (Ni/Ti) thin bilayers were deposited on the surface of the polymer helical micromachine by electron beam (e-beam) evaporation for magnetic actuation and improvement of surface biocompatibility. Scale bars: 10 μm and 2 μm. Reproduced with permission from [41], Copyright © 2012, Wiley. (**b**) Time-lapse photo of the controlled actuation of a functional artificial bacterial flagellum (f-ABF) to the target cell (cell 3, the contour is circled in red). Cell 3 was under cell division and was considered as one single cell in this case. Cell 2 and cell 4 are blurred in the image due to the drift of cells in the medium during the time-lapse image. The movement of the f-ABF is marked with blue rectangles. The interval of each movement was 4 s. The inset shows f-ABF in contact with cell 3. Reproduced with permission from [69], Copyright © 2015, Wiley. (**c**) 3D printed microcapsule (top) and microsyringe (bottom) functioning with the same reciprocating mechanism. Such a microcapsule can be used for spatiotemporally controlled delivery of therapeutic agents driven by magnetic field. The scale bar is 20 µm. Reproduced with permission from [70], Copyright © 2016, American Chemical Society. (**d**) Design, fabrication, SEM image, and magnetic control of 3D-printed microrobotic transporters. Scale bars: 10 μm. Reproduced with permission from [71], Copyright © 2019, Wiley. (**e**) Higher forward swimming capability of conical microhelices. Schematic of the actuation scheme, where M is the magnetic dipole moment of object, B is the external magnetic field, and T is the magnetic moment. Scale bars: 50 μm. Reproduced with permission from [72], Copyright © 2019 Wiley. (**f**) Images by fluorescence microscopy show the microtube rotation with the rotation of an external magnetic field. Time-lapse images show the motion of microtubes. Under the propelling and guiding of external magnetic field, a specific route of pattern “HI” is realized. Reproduced with permission from [73], Copyright © 2019, Wiley.

Zhang and Nelson et al. designed and fabricated magnetic helical swimmers using 3D direct laser writing (DLW) and physical vapor deposition (Figure 2a) [41]. These devices achieved corkscrew motion in a fluid via the relatively high frequency of the rotating field. After demonstrating excellent swimming performance in water and fetal bovine serum (FBS), these helical micromachines were employed to transport microparticles. These helical swimmers can perform 3D navigation in a controllable fashion with micrometer precision in low-strength rotating magnetic fields (<10 mT), and they are promising tools for targeted drug delivery in vitro and in vivo. This strategy is so universal and reliable that a series of research studies were performed following this work. Qiu further improved this approach by functionalizing with lipoplexes to enhance the biocompatibility of these types of helical swimmers. Subsequently, the successful wirelessly targeted single-cell gene delivery to human embryonic kidney (HEK 293) cells using artificial bacterial flagella (ABFs) loaded with plasmid DNA (pDNA) in vitro was demonstrated for the first time (Figure 2b) [69]. This work presents the possibility of such magnetic swimmers targeting hard-to-reach areas in the human body, since the flagellum-propulsive method is a promising approach in viscous liquid (e.g., blood and interstitial fluid). Similarly, Schmidt’s group customized microhelices to serve as motors for transporting sperm cells with motion deficiencies to help the cells carry out their natural function. They mimicked such motors in in vivo conditions and applied hypoosmotic swelling as a method for sperm selection in a microfluidic channel platform. Although the complete release of the sperm cell after motion was not successful due to the unspecific adhesion of the microhelix (Figure 2c), this magnetic–biological hybrid propulsion exhibited prospects to cure cell diseases [70].

To improve the clinical outcomes, efforts should focus on the active delivery of stem cells to a target tissue site within a controlled environment, increasing the survival rate of effective tissue regeneration. Yasa et al. presented a remotely steerable microrobotic cell transporter with a biophysically and biochemically recapitulated stem-cell niche for directing stem cells toward a predestined cell lineage (Figure 2d) [71]. The microrobotic cell transporter was fabricated through two 3D printing steps with TPP. The first step involved the alignment of nanoparticles radially with the continuous application of a uniform external magnetic field during the fabrication process, while the second step showed the biological niche construction within the MCT inner cavity. The magnetically actuated double-helical cell microtransporters with 76 µm length and 20 µm inner cavity diameter were 3D printed, and biological and mechanical information regarding the stem-cell niche was encoded at the single-cell level. Cell-loaded microtransporters were mobilized inside confined microchannels along computer-controlled trajectories with rotating magnetic fields. The authors expect that such microrobotic devices have the potential to enable the development of active microcarriers with embedded functionalities for controlled and precisely localized therapeutic cell delivery.

Advanced fs-laser printing has been used for efficient fabrication with spatial light modulation (SLM) technology. Xin et al. manufactured conical hollow microhelices with designable heights (H = 45–75 µm), diameters (D = 6–18 µm), pitch numbers (Pi = 2–4), taper angles (T = 0.1–0.6 rad), and pitch periods (ΔP = 10–30 µm) to improve the performance of magnetic controlled motion [72]. Compared with straight microhelices, the forward swimming capability of the conical microhelices was increased by 50%, and the lateral drift of the conical hollow microhelices was reduced by 70% (Figure 2e). Additionally, the one-step synthesis of 3D biconical microtubes with the single exposure of a femtosecond optical vortex was achieved by Yang et al. [73] These biconical microtubes present unique advantages over straight microtubes in terms of the confinement and transportation of microcargo, which can prevent the cargo from falling off during transit because of fluid impact. The authors performed an in situ cell experiment related to the development of doxorubicin (DOX) in targeted Hela cells and the treatment effect for exhibiting the potential of such tubular microactuators in single-cell analyses and drug screening (Figure 2f).

### 2.2. Light

Light plays a very important role in both the manufacturing and the manipulation of microstructures [57,74,75,76]. Light is a pure and safe energy source that can be transmitted to target objects in a noncontact manner, which enables remote control [77,78,79,80]. It is well known that optical tweezers have been widely used to manipulate objects of different sizes, a process which received the Nobel prize in 2018. Light-induced driving is based on a variety of photochemical/photothermal mechanisms [81].

Direct light-driven microactuators suffer from low efficiency in that converting light to mechanical power detracts from its advantages because the light force is too small to drive a structure at the scale of tens of microns. In a systematic study on conversion efficiency, Lin et al. designed a turbine-like micro-rotor and carried out a quantitative analysis with computational fluid dynamics and semiclassical optics [82]. The motor was designed as a spiral phase plate (SPP). When a plane wave passes through a SPP, it is converted into a helical wave that carries orbital angular momentum. At the same time, this gives the SPP an inverse angular momentum, exerting a torque that may actuate a rotation of the SPP. By optimizing the shape design, this rotor presents a very large rotation speed (over 500 r/min). Denoted by the average angular momentum transfer, the rotor’s light conversion efficiency has been experimentally determined to be as high as 34.55.

**Figure 3:**
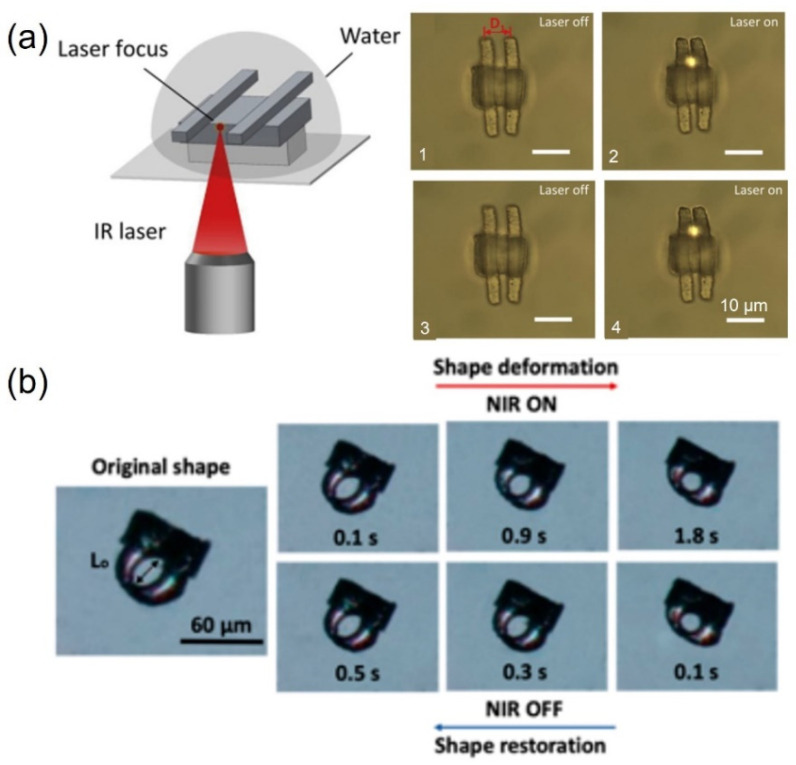
(**a**) Scheme and optical microscope photographs of the NIR laser-driven hydrogel microactuator. Reproduced with permission from [83], Copyright © 2020 Elsevier. (**b**) The shape deformation and restoration of a light-controllable microgripper under NIR phase transition. Reproduced with permission from [84], Copyright © 2019, American Chemical Society.

The development of light-driven microactuators is hampered due to the difficulties in designing and fabricating biocompatible light-driven microactuators with dimensions of less than 100 µm and a response time on the order of seconds. Some nanoparticles (NPs) doped in materials can efficiently absorb light and produce heat, resulting in thermal deformation for a good strategy. Researchers have reported a light-triggered microactuator composed of photothermal Fe_3_O_4_ NPs, with an average size of 7.7 nm, and a thermally responsive crosslinked PNIPAM hydrogel [83]. A double-armed near-infrared (NIR)-light-driven three-dimensional (3D) hydrogel microcantilever with a size of ~26 μm was successfully fabricated. (Figure 3a). With NIR radiation, the hydrogel microactuator in water presents a fast response time of ~33 ms, which is the fastest light-responsive hydrogel microdevice yet reported [83].

Using the same idea of doped photothermal materials, Chen et al. mixed gold nanorods (AuNRs) in liquid crystal elastomers (LCE) (Figure 3b) [84]. They enhanced the miscibility between the AuNRs and the LCE with thiol functionalization and they studied the appropriate mixture to increase the mechanical properties and near-infrared (NIR)-responsive mechanical deformation. The effects of the AuNR loading fraction and the laser power on the light-powered actuating performance were evaluated. The authors found that the nanocomposite with AuNR loading of 3 wt.% demonstrated the maximum percentage (20%) of elongation for an NIR laser power of 2 W. They fabricated a microgripper that presented a rapid light driven deformation. The gripper shrank in 2 s with the irradiation and opened again 0.5 s after removing the NIR.

### 2.3. pH

pH-responsive materials have drawn researchers’ attention because of their easy integration with microfluidics or liquid environments. For example, a pH-sensitive hydrogel composite could be regarded as an artificial musculoskeletal system for TPP printed microgrippers [50].

We developed a photosensitive stimulus-responsive hydrogel precursor composed of acrylic acid (AAc), *N*-isopropylacrylamide (NIPAAm), polyvinylpyrrolidone (PVP), dipentaerythritol hexaacrylate (DPEHA), triethanolamine (TEA), and 4,4′bis(diethylamino)benzophenone (EMK). TEA acts as the photosensitizer to help the photoinitiator EMK to produce radicals with the excitation of the femtosecond laser. With the assistance of free-radical polymerization of the acrylate group, a linear copolymer between AAc and NIPAAm monomers is formed. At the same time, the radical also activates crosslinker DPEHA to interconnect the poly (AAc-*co*-NIPAAm) chains and form the hydrogel framework. It should be noted that the crosslinking density should be controlled in a relatively low level in order to achieve a large swelling ratio. By adding PVP in materials, the mechanical properties of the polymerized hydrogel are increased to constitute 3D freestanding structures. The critical pH value of such a prepared material is 9. By utilizing such prepared pH-sensitive hydrogels, microstructures can be conveniently adjusted by varying the pH.

**Figure 4:**
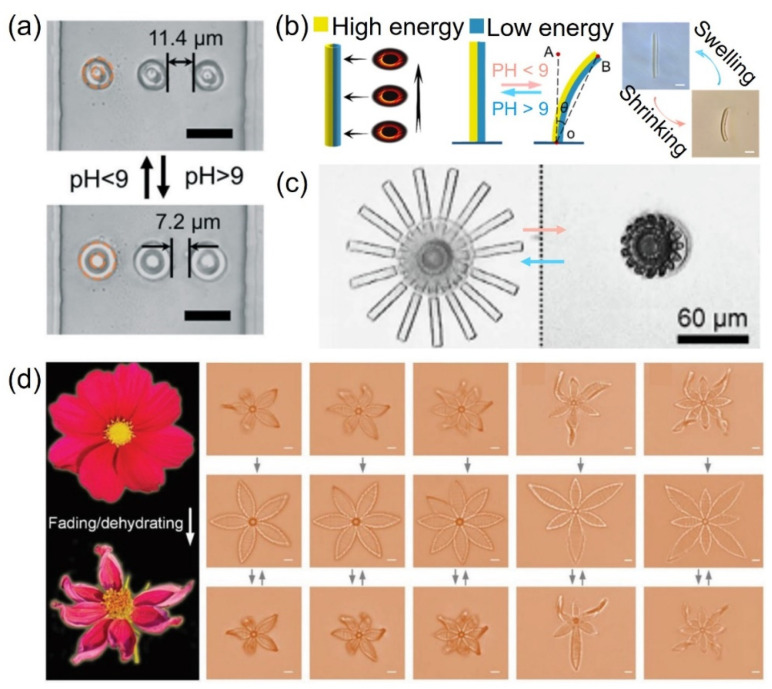
(**a**) The matryoshka-ring filter for multi-filtering of particles with different sizes by changing the pH. Reproduced with permission from [85], Copyright © 2019, Royal Society of Chemistry. (**b**) pH-responsive actuator fabricated by dynamic asymmetric femtosecond Bessel beam. Reproduced with permission from [86], Copyright © 2020, American Chemical Society. (**c**) Complex 3D micro-umbrella, which can be constructed to achieve rapid, precise, and reversible articulated-lever folding by varied pH value. Reproduced with permission from [24], Copyright © 2020, Elsevier. (**d**) Botanical-inspired complex shape transformation and on-demand microparticle capturing. The withering process of flowers in nature. Various bending blade structures mimicking the flowers prepared with different laser power. Reproduced with permission from [87], Copyright © 2019 Wiley.

Trapping methods based on microfluidic chips are an efficient method for single-particle/cell isolation due to their low reagent consumption, fast sample processing, high integration, portability, and low cost. However, in many single-particle/cell capture arrays integrated in microfluidic devices, a constant pressure is always required to prevent the captured particles from escaping, which limits the applications of these devices. In order to solve this problem, Hu et al. integrated a pH-sensitive microring array into a “Y”-shaped microfluidic channels [85]. In this research, these uniform ring structures were able to swell and shrink in less than 200 ms with the change in environmental pH value. The pH-responsive properties of the hydrogel can be precisely controlled using different laser processing dosages including swelling ratio (35–65%) and diameter change (2–5 μm). The tunable microfluidic device demonstrated its ability in multi-filtering polystyrene (PS) particles and in completely trapping PS particles with a specific size. As shown in Figure 4a, the distance between two adjacent rings can be varied from 11.4 μm to 7.2 μm such that objects with specific sizes can pass through or be intercepted selectively.

As described in the above article, the exposure energy on the ring is uniformly distributed. Since the swelling factors of the hydrogels are closely related to the exposure dose, programming such a dose spatially on the Bessel ring leads to facile fabrication of anisotropic structures. Li generated a heterogeneous light field during the fabrication process, resulting in microtubes with asymmetric swelling/shrinking properties that exhibited reversible bending and reverting to an upright conformation within 1 s when the solution pH value changed (Figure 4b) [86]. He also demonstrated the application of this type of effective microgripper for trapping and releasing PS particles and neural stem cells.

Although bending, folding, and twisting of stimulus-responsive structures have been presented by a large number of studies, the construction of 3D structures at a small scale with a high shape-morphing freedom poses challenges. Jin et al. utilized the flexibility of TPP and the adjustability of pH-hydrogels to construct large-scale complex reconfigurable structures with arbitrary 3D shapes and sub-micrometer features [24]. A foldable micromachine that could morph in a similar way to the opening and closing of an umbrella was printed (Figure 4c). This compound micromachine contained 15 deformable stretchers that were distributed in a circle at regular intervals on the shaft periphery. Each stretcher was a thin bilayer structure, and it was connected to a thick umbrella rib. Similarly, biomimetic microscale hydrogel structures on the order of 10 μm were created by Hu et al. [87]. They found that both the exposure dose and the scanning direction could change the response properties of these hydrogel microactuators. By introducing localized nonuniform defects in the 3D microstructures, more complex shape-morphing behaviors can be expected because the relative expansion ratio can be versatilely distributed. To this end, a femtosecond laser (~80 fs, 80 MHz) was employed for hydrogel structuring. Due to the fluctuance of the laser output and the fluidity of the hydrogel, blade structures with local nonuniformity were produced using low laser power and low scanning repetition. Moreover, the microstructures appeared to be very soft due to the weak crosslinking of hydrogel polymer chains, making complex 3D shape morphing much easier. Microparticles were used for the proof-of-concept demonstration of the on-demand capture and release functionalities of the pH dominated micro-architecture via a complex deformable microcage, whose shape was similar to that of a football.

However, the pH level needed to initiate this shape change for our material was greater than 9. This environment would negatively impact cellular health and may even kill the cells that a scientist is attempting to “manipulate”. In addition, adding acid dropwise to a solution housing cells and/or biological proteins can greatly impact the viability/function of the cells. Therefore, in future studies, developing materials with a milder or tunable pH threshold is one potential solution.

### 2.4. Microforce

A force that can act at the microscale level is also an effective driving force for a microactuator. Capillarity is common both in daily life and in micro/nano fabrication when a solid–liquid interface exists. The capillary force tends to dominate over the standing force of microstructures, and this needs to be overcome for coalescence when the scale is reduced. When one aims to create slender structures in micro/nanoelectromechanical system manufacturing processes, the dominant capillary force drives the structures to collapse, cluster, or be completely destroyed. Some efforts, such as adopting a supercritical-point dryer, have been made to eliminate these unwanted behaviors when fabricating high-aspect-ratio structures. However, from another point of view, the capillary force can be exploited as a valuable tool to drive microstructures. Compared with the other driving forces of microactuators such as magnetism, pH, and light, capillary force features the advantages of simplicity, low cost, scalability, and tunability [88,89,90].

**Figure 5:**
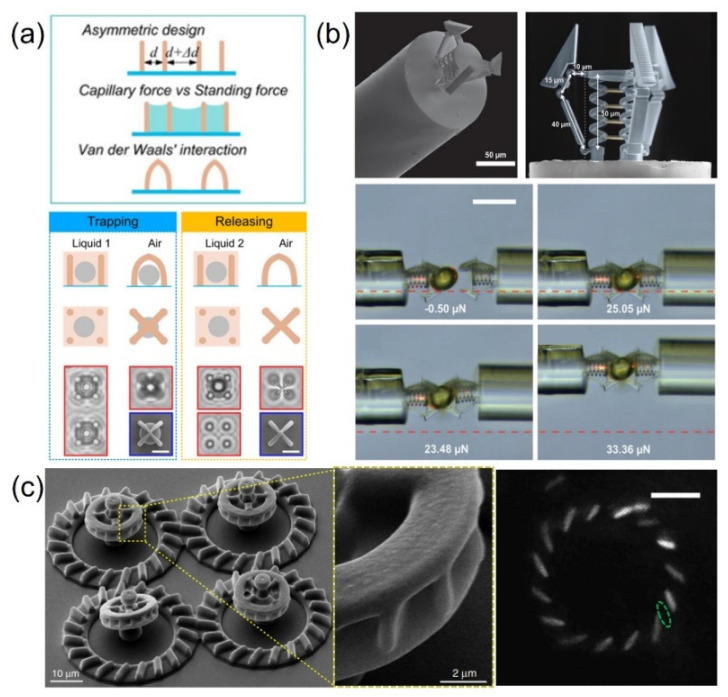
(**a**) Capillary-force-driven microactuator and its application for microparticle gripping. Reproduced with permission from [91], Copyright ©2015, National Academy of Sciences. (**b**) Monolithic force-sensitive 3D microgripper fabricated on the tip of an optical fiber using two-photon polymerization. Reproduced with permission from [93], Copyright © 2018 Wiley. (**c**) Bacteria-driven micromotor. Reproduced with permission from [94], Copyright © 2017, the authors.

The combination of TPP and capillary force can be used to construct microgrippers for the selective trapping and releasing of microobjects, which are intensively demanded in the fields of chemical analysis and biomedical devices and which have drawn considerable attention from researchers. The concept of this type of capillary-force-driven gripper is sketched in Figure 5a [91]. Pillars with designable diameters, heights, and positions have been printed with TPP to adjust the capillary force and standing force during the evaporation of the developer. If the capillary force is larger than the standing force, pillars will congregate with adjacent pillars to form a gripper. In the experiment, the maximum capillary force was calculated to be about 2 μN. The van der Waals force (Fv) between assembled pillars resists the standing force and keeps the gripper closed. More interestingly, when the assembled grippers are immersed in liquid again, the Fv decreases such that the standing force of the pillars dominates the pillars’ restoration to an upright position, which makes the gripper open again. Therefore, selective trapping and releasing can be achieved, as shown in Figure 5a. This artificial microgripper is capable of capturing microparticles with excellent selectivity. The particles can be trapped inside the “cage-like” spaces constructed from four bent pillars by the capillary force. Furthermore, the size of the particles to be captured can be controlled by adjusting the intracell pitch of the assemblies. Only those particles of suitable size can be effectively caught due to the space limitation of the microgripper. Due to the design flexibility of this laser printing-induced capillary force self-assembly technique, the intracell pitch between adjacent pillars can be easily changed to trap specific particles. To further investigate the trapping ability of the artificial microgripper, the authors prepared a series of assemblies to trap various particles, with sizes from 1 μm to 4.6 μm. Microparticles with different sizes can be trapped by tuning the intracell pitches accordingly. The as-prepared grippers could be reused, and they exhibited no obvious functional degradation after over a dozen repetitions. Microgrippers with the selected particles have been immersed into another solvent (e.g., acetone). The grippers open and the selected particles are released into the new solvent. This capillary-force-driven gripper can also be manufactured on soft PDMS such that the size of the trapped particles can be adjusted on demand with a mechanical stretching method [92]. This ability for microobject selective transportation may find wide applications due to its scalability, designability, and reproducibility.

Although many grippers fixed on a base can trap microobjects with good selectivity, the trapping is passive. To actively capture objects, a mechanical force-driven gripper that can be positively manipulated is an ingenious candidate. Power et al. presented a tethered, 3D, compliant gripper with an integrated force sensor, which was fabricated on the tip of an optical fiber in a single-step process using TPP (Figure 5b) [93]. The passively actuated grasper measured approximately 100 µm in length and breadth, while its position could be controlled via the micromanipulation of the optical fiber. The force-sensing elements were designed using optical interferometry and embedded in the structure. Therefore, the force estimation could be measured in real time. They also demonstrated the manipulation capabilities and tested the real-time force-sensing capability of the passively actuated microgripper with an accuracy below 2.7% of the maximum calibrated force. As shown in the optical microscope images in Figure 5b, a solid ellipsoidal object with dimensions of 50 µm × 50 µm × 70 µm could be grabbed, transferred, and released by two identical microgrippers. The force of mechanical gripper varies between 10 and 50 μN. Benefiting from the universality of trapping without considering the physical and chemical properties of the target object, this gripper is useful for the study of biological structures such as alveoli, villi, or even individual cells. More interestingly, the authors skillfully combined the force feedback system via a high-dimensional spectral reading with artificial neural networks to predict the axial force exerted on/by the gripper, which could play a big role in smart medicine in the future.

In addition to capillary forces and mechanical forces, microorganisms (e.g., bacteria) have also been recruited to push microactuators. However, the force of an individual microbe is too small to propel a microactuator, while the work of a large number of microbes cancels out with each other, resulting in the fact that the proposed designs of self-propelled bacteria suffer from low reproducibility, large noise levels, or a lack of tunability. To overcome this issue, Vizsnyiczai et al. demonstrated a fast, reliable, and tunable bio-hybrid micromotor that could be obtained via the self-assembly of synthetic structures with genetically engineered biological propellers [94]. As shown in Figure 5c, the structure had three component parts. The rotating unit (appearing in green) had an external radius of 7.6 mm and a thickness of 3.7 mm. Its outer rim featured 15 microchambers that could each capture individual bacteria. The zoomed-in view of these microchambers shows the tilted angle (which was 45°) for adjusting the direction and the number of captured bacteria so that the maximum torque could be achieved. The cell bodies in the micromotors could be clearly seen via fluorescence microscopy, which was fitted with an ellipsoidal shape, shown as a dashed line in Figure 5c. By using the phototaxis of the bacteria, the rotation speed of the bacteria-propelled micromotors could be controlled by light, which provided a solution to increase the controllability of the microbe driven micromotors. The bacteria-driven micromotor presents an effective pushing force per cell that is 0.2 pN. In addition to the bacteria, bovine sperm cells have also been utilized to drive micromotors made by TPP [70]. However, the survival of microbes or cells requires the presence of specific nutrients and suitable pH in the surrounding environment, which may reduce the generality of microbe driven actuators. Although some problems still remain for microbe-propelled micromotors, such a strategy also provides a method for constructing hybrid actuators.

### 2.5. Heat

Thermal deformation is a common physical phenomenon, and it can be used to drive microactuators based on heat-sensitive materials that can deform significantly under heat stimulation [95].

**Figure 6:**
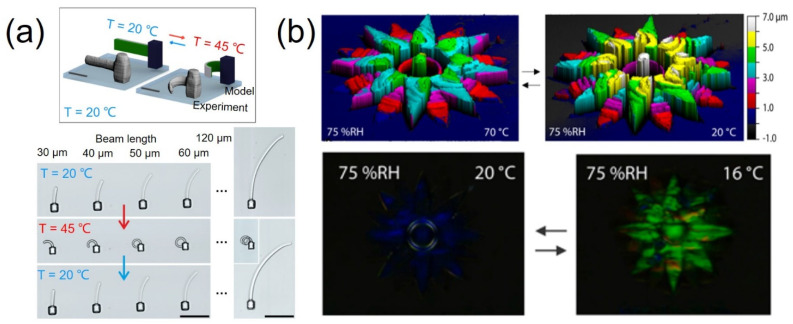
(**a**) 3D TPP pNIPAM-based hetero-microstructures for heat-driven microactuator and the temperature dependence of five structures with different beam lengths prepared under identical fabrication conditions. Scale bars are 20 µm and 50 µm. Reproduced with permission from [96], Copyright © 2019, the authors. (**b**) 3D profiles and crossed polarized micrographs of the flower that depict indirect (triggered by heat) actuation. RH: relative humidity. Reproduced with permission from [97], Copyright © 2020 American Chemical Society.

Poly(*N*-isopropylacrylamide) (pNIPAM) is a well-established polymer that exhibits a substantial response to changes in temperature close to its lower critical solution temperature. Hippler et al. developed functional three-dimensional hetero-microstructures based on pNIPAM to create actuation patterns that could react to different stimuli [96]. By adjusting the exposure dose during TPP, they altered the material-response parameters on demand, resulting in large property differences. Figure 6a shows the scheme of the bimaterial heterostructures with the two parts highlighted in green and gray being the lower and higher dose exposures, respectively. The beams made with one material and processed with different exposure doses started straight at T = 20 °C and became curved at T = 45 °C.

More complex 3D heat-responsive microstructures can be achieved with TPP. Del Pozo et al. presented a supramolecular cholesteric liquid crystalline photonic photoresist for the fabrication of 4D photonic microactuators such as pillars, flowers, and butterflies with submicron resolution [97]. They employed a photonic photoresist based on a hydrogen-bonded cholesteric liquid crystal (CLC) mixture that could be polymerized into coatings to demonstrate structural color and shape changes. A slice thickness of 0.5 μm was used to fabricate the 6 μm tall flower to exhibit the heat-responsive photonic microactuators. When the structure was placed in a thermal environment, water in structures evaporated such that the height of structure changes was affected by the environment temperature and the humidity. Figure 6b showcases the obvious changes in the 3D profile (upper) and structural color (bottom) triggered by different temperatures. It is worth noting that the heat-response change can also be achieved by adjusting the temperature of the structure.

### 2.6. Solution

Catalytic bubble propulsion is one of the major actuation methods in solutions, which mimics macroscopic rocket propulsion [98,99,100]. Solution-driven microactuators can convert chemical energy from a surrounding aqueous fuel solution into mechanical energy to generate autonomous movements. The most common chemical reaction used in catalytic self-propulsion is the decomposition of H_2_O_2_ under the action of a metallic layer such as Pt or Ag. For example, self-assembled tubular micro/nanomachines and Janus micro/nanoparticles propelled by oxygen bubbles decomposed by hydrogen peroxide have been proven by researchers.

**Figure 7:**
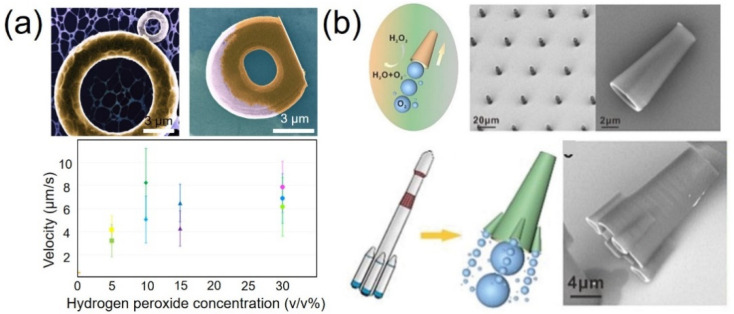
(**a**) TPP printed doughnut swimmer. Upper panel: High-resolution transmission electron microscope (HRTEM) and SEM image of doughnut simmer with two different surface coatings. The bottom of the right swimmer is “cut off” at the bottom to provide a stable base during printing and the metal evaporation. Lower panel: A graph representing the propulsion velocity dependence on the concentration of hydrogen peroxide. Scale bars: 3 μm. Reproduced with permission from [101], Copyright © 2019, Springer nature. (**b**). Schematic diagrams of fabrication of tubular micro/nanomotors. Upper panel: SEM image of an array of microtubes on glass substrate fabricated by 3D-DLW lithography. Lower panel: Model of a rocket in the macro world, showing schematic diagram of a tubular microrocket and SEM images of tubular micro/nanomotors with a similar structure to rocket boosters inspired by a macro rocket. Reproduced with permission from [102], Copyright © 2019 Wiley.

Remmi et al. used TPP to fabricate multi-responsive artificial microswimmers [101]. Doughnut-like swimmers were fabricated and coated with platinum catalytic layers so that they could be actuated by chemical power. In water, the tori exhibited pure Brownian diffusion. However, in the presence of hydrogen peroxide, the 7 μm doughnut consumed the fuel and directionally translated across the surface. The propulsion velocity of the tori scaled linearly with the concentration (*v*/*v*%) of hydrogen peroxide up to 10% and then saturated from 10% to 30% (Figure 7a). In 30% hydrogen peroxide, the doughnut swam with a speed of about 7 μm/s. Such shape-programmed microswimmers have the potential to manipulate biologically active matter such as bacteria or cells.

Chen et al. fabricated highly efficient chemical catalytic microtubular motors via 3D laser lithography and demonstrated their motion behavior with the driving force coming from the bubble produced by catalysis (Figure 7b) [102]. By adjusting the geometrical shape and the surrounding chemical fuel environment of the micro/nanomotor, the frequency of the catalytically generated bubble ejection could be tuned, resulting in the variation in the motion speed. Compared with a single tubular or Janus particle micro/nanomotor, such micro/nanomotors presented an enhancement in speed benefiting from their rocket-like shape.

### 2.7. Acoustic Field

Acoustic equipment such as B-scan ultrasonography is widely used in medical clinical diagnosis due to its biopenetrability. By combining the acoustic method with microfluidics, Huang’s group developed a series of creative strategies for important applications in analytical chemistry and biomedicine, including cell separation, sample enrichment, fluid pumping, reagent mixing, on-chip PCR, biophysical measurements, and the manipulation of model organisms [103,104,105,106]. Acoustic field-driven nanomotors have been demonstrated for the reversible assembly of catalytic nanomotors, controlled swarm movement, and the separation of different nanomotors [107,108]. However, current acoustic-driven micro/nano motors or swimmers are always nanoparticles that possess simple shapes. At present, the research about acoustic technology focuses on group control, but the design of the acoustic actuator itself is rare.

**Figure 8:**
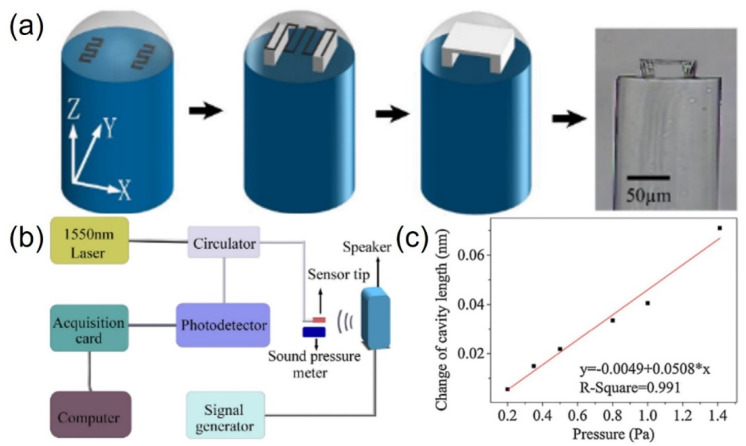
Actuators which can transfer acoustic waves to mechanical movement, resulting in (**a**) actuators made by TPP on fiber. (**b**) Schematic diagram of experimental setup for acoustic pressure measurement. (**c**) The measured relationship between the change in cavity length and the acoustic pressure at 1 kHz. Reproduced with permission from [109], Copyright © 2017 Elsevier.

Li et al. fabricated an acoustic sensor that could transfer an acoustic signal to the mechanical movement on an optic fiber [109]. The fiber sensor tip was fabricated with TPP and it consisted of two sidewalls on the end surface of the single-mode fiber and a film held up by the sidewalls, which formed a fiber inline Fabry–Pérot microcavity (Figure 8). The quadrature phase bias point was controlled at a fixed wavelength of 1550 nm by accurately matching the cavity length with the film thickness; the corresponding acoustic pressure of the fiber sensor tip reached 0.0508 ± 0.0052 nm/Pa. A fiber acoustic sensor tip can be suitable for both industrial and biomedical applications due to its high sensitivity, excellent biocompatibility, ultracompact size, and independence of laser wavelength tuning. Although such a device is more like a sensor than a microactuator, this work also provides a reference for 3D acoustic actuators fabricated with TPP or other 3D printing methods.

### 2.8. Electric Field

It has been about 150 years since the second industrial revolution, when humans entered the age of electricity with the invention of the electric generator and electric motor. Given the universal applications of integrated circuits at the single nanoscale, an electric-driven device can be considered as another type of powerful microactuator that has huge potential for miniaturization [110]. Conventionally, electrical-driven components are based on rigid devices (e.g., coil-based electromotors and piezoelectric actuators), which hinders the miniaturization of electrical actuators [111].

Recently, electroactive smart materials (e.g., hydrogels) have exhibited large deformation in response to an electric stimulus and they have received significant attention as a potential actuating material for soft actuators and artificial muscle [112]. Electric-activated materials are always rich in ions that can re-distribute in electrical fields in an electrolyte solution, so that structural deformation can be achieved. Although there are few reports involving TPP being used for electric stimulating materials according to our best knowledge, some interesting work about electric-driven soft actuators has been done by researchers using the laser direct writing method.

**Figure 9:**
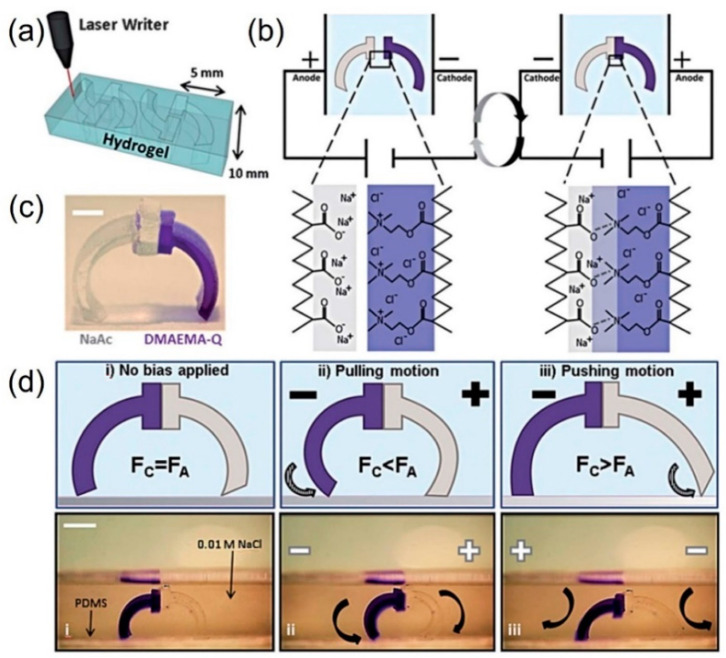
Electric-driven hydrogel actuators. (**a**) Laser cutting process. (**b**) Actuation mechanism of the hydrogel actuators. (**c**) Photograph of a finished gel walker. Scale bar: 2.5 mm. (**d**) Illustrations and photographs of a gel walker composed of NaAAc and 30% DMAEMA-Q legs in 0.01 M NaCl with an applied field of 5 V/cm. Reproduced with permission from [113]. Copyright © 2014, Royal Society of Chemistry.

Employing gel actuators composed of cationic and anionic gel legs made of copolymer networks of acrylamide (AAm)/sodium acrylate (NaAAc) and acrylamide/quaternized dimethylaminoethyl methacrylate (DMAEMA Q), Morales et al. presented a gel walker that achieved unidirectional motion on flat elastomer substrates in NaCl aqueous solutions (Figure 9) [113]. They studied the relationship between the mechanical behaviors of this electric-response actuator and the environmental parameters, and they found that the most responsive gel system contained the most ionized fixed charges (highest effective charge density, as opposed to the highest amount of NaAAc) in a dilute salt solution to maximize the osmotic bending force, which was governed by the mobile ion difference between the gel and the solution. When the external solution lacked mobile ions, bending under electric stimulus was negligible; when the amount of overall fixed charges in the gel network was too high, mobile ions condensed on the polymer backbone, leading to the decrease of bending. These electric-driven actuators can be used for cargo transport. Yang et al. developed soft hydrogel walkers made with a mold-assisted strategy and studied their driven motility [114]. The hydrogel walkers consisting of polyanionic poly(2-acrylamido-2-methylpropanesulfonic acid-*co*-acrylamide) exhibited an arc looper-like shape with two “legs” for walking. The hydrogel walkers could reversibly bend and stretch via repeated “on/off” electric triggers in the electrolyte solution. Based on such bending/stretching behaviors, the hydrogel walkers could move their two “legs” to achieve a one-directional walking motion on a rough surface via repeated “on/off” electric triggering cycles. Moreover, the hydrogel walkers loaded with very heavy cargo also exhibited excellent walking motion for cargo transport.

Nevertheless, the mechanical actuation of electric-driven actuators has always been limited for simple bending or folding due to uniform electric field modulation. To implement complex movements, actuators always require a pre-program, such as a hinge and a multilayer pattern, which will increase both the number of steps and the difficulty of fabrication. Choi et al. proposed a reprogrammable actuating method and sophisticated manipulation by using multipolar three-dimensional electric field modulation [115]. The polarity/intensity of the electric field in three dimensions (3D) could be adjusted through the multipolar spatial electric field modulator so that the complex 3D actuation of single hydrogels was achieved. This method also prevented the issue of air bubbles being generated during operation in the conventional horizontal configuration.

Although millimeter-scale electric-driven actuators have been achieved, electric driving at the microscale remains challenging. Since electrically driven materials need enough volume to hold enough ions to form anisotropy under electric field stimulation, more sensitive electric activating materials and more flexible processing methods are needed for microscale electric-driven soft actuators. We think that TPP and hydrogels possess potential in this area.

### 2.9. The Comparison of Actuation Methods for 3D Microactuators

As presented, there are a variety of actuation methods for triggering 3D microactuators. Given that each method has its own advantages and disadvantages, it is necessary to summarize and discuss each method (Table 2). Depending on whether contact is required, the driving methods can be divided into two categories: external field-driven methods and contact driving methods. The former includes magnetic fields, light, heat, acoustic fields, and electric fields, while the latter involves pH, microforces, and solutions.

External field-driven strategies provide wireless, remote, and noncontact manipulation strategies. Currently, the most popular external field-driven methods include magnetism, acoustics, and light, which have their strengths and weaknesses.

Both magnetic and acoustic fields can be employed for collective manipulation. Additionally, they all have outstanding biological penetration and safety, making them ideal tools for medical applications of 3D microactuators. However, magnetic fields can be used for 3D space manipulation, while sound fields are generally used for 2D horizontal manipulation. Therefore, during recent work with 3D microactuators, magnetic driving demonstrated better controllability than acoustic triggering.

Compared to magnetic and acoustic driving, light triggering provides unique advantages beyond collective and remote manipulation. Firstly, benefiting from microoptical technology, the control accuracy of light manipulation, which includes both the moving route and the position, is much higher than that of magnetic and acoustic driving. Secondly, light driving also enables individual manipulation for a group of targets, which is not possible with magnetic and acoustic fields. Lastly, the most important factor is that light possesses more adjustable parameters and, hence, excellent programmable properties. For example, light with different wavelengths can simultaneously manipulate nearby objects without the wavelengths interfering with each other. However, the poor biological penetration greatly limits the practical application of the light driving method.

Compared with external field-driven control, the contact control method, without complex equipment to maintain the external energy field, is usually simple and convenient. For example, pH and solution driving only requires dropping the corresponding liquid on the microactuators. This advantage makes it easier to use a contact-driven 3D microactuator in combination with other micro/nano techniques (e.g., microfluidics), and the advantage is conducive to its miniaturization. For example, pH-triggered microactuators with sub-micron diameters can be achieved in microchannels with the marriage of TPP and pH-sensitive hydrogel [116]. The main obstacle for the wide application of these pH and solution driving methods is poor biocompatibility. Specifically, most liquids (e.g., a solution with alkaline pH or hydrogen peroxide) employed for driving are toxic to living cells. As for microforce manipulation, the contact mode also hinders its wide applications.

## 3. Discussion and Summary of TPP for Microactuators

Although a large amount of impressive work has been done regarding the fabrication and application of 3D microactuators via TPP (Table 3), there is still an abundance of room for improvement in terms of materials, design and machining, and driving modes to facilitate practical applications [14,117,118,119].

### 3.1. Material

In addition to traditional polymer photoresists used for TPP, researchers have developed more functional materials, as well as processing strategies, for 3D laser direct writing [117].

#### 3.1.1. Doped Photosensitive Polymer

At present, photosensitive polymers are the main materials for TPP, and these polymers can be roughly divided into two types: free-radical triggering and cationic triggering. In order to extend the capabilities of the two-photon printing of 3D structures, researchers have tried doping functional materials (especially inorganic materials) into polymer materials, such as metal nanoparticles, carbon nanotubes/wires, and graphene [120]. Compared to pure polymer photoresists, doped photoresists have a lower laser intensity threshold and a higher critical laser scanning speed due to the high absorption of doped functional materials at near-infrared wave light, which enhances the photosensitivity of a photoresist. Therefore, a shorter printing time can be achieved for a doped photoresist.

Inorganic materials will greatly expand the functions of microrobots and open up new applications. For example, full Fe structures are a very promising biocompatible approach for applications requiring enhanced magnetic performance in the field of medical microrobotics [121,122,123,124]. Currently, some strategies (e.g., fused deposition modeling, selective laser sintering, and ink-jet printing) have been used to prepare the microstructures of inorganic materials such as ceramics, metals, glass, and liquid metals. However, the fabrication of true 3D inorganic structures at the micron scale is still challenging. Furthermore, microactuators always require movable components or compliant joints for shape transformation or advanced functionalities, which are more easily achievable using soft structures. The doping of inorganic nanoparticles in a photoresist has proven to be a useful strategy to form printing material that can be used for TPP.

However, doping may lead to a homogeneity problem caused by the mixing inhomogeneity and anisotropy of functional materials. In addition to the problem of mixing, another problem is that the large thermal changes caused by light absorption may hinder the formation of three-dimensional structures. For example, during the writing process of a graphene oxide (GO) photoresist, excessive energy absorption by GO led to tremendous heat generation and, thus, vigorous bubble formation, which in turn hindered the formation of the 3D structure. There are some other issues to be conquered: (1) light scattering caused by refractive index differences between doped inorganic materials and photosensitive resins, and (2) stress and adhesion between doped inorganic materials. To address such problems, some interesting work has been done. For example, De Marco et al. developed a TPP-assisted casting strategy with which any water-soluble material could be shaped into almost any arbitrary shape with feature sizes in the 5 μm range [125]. The method ensured processing compatibility between metal and polymer 3D molding at the microscale and simplified the manufacturing steps. We believe that inorganic materials that can be used for TPP will be applied in more fields.

#### 3.1.2. Liquid Crystal Elastomer

Liquid crystalline elastomers (LCEs) are soft materials that contain mesogenic moieties with a self-organization capability. They are another promising type of material for constructing microactuators [22,126]. These materials can respond to external stimuli such as heat with a macroscopic shape change due to a nematic-to-isotropic phase transition of the mesogens [127]. This reconfigurable property of LCEs is promising for the development of advanced actuating, sensing, and robotic devices. By doping photoinitiators, crosslinkers, and nanocomposites containing photothermal nanomaterials including multiwalled carbon nanotubes, graphene oxide, and gold nanorods (AuNRs), researchers have developed photosensitive LCEs for TPP DLW.

#### 3.1.3. Shape Memory Polymer

A shape memory polymer (SMP) is a “smart” material that can recover its initial shape through external stimulation (such as heat or electricity) [128]. Although an SMP does not directly respond to laser light, the induced heat by a laser will alter the morphology analogously to naturally occurring heliotropism [1]. Preliminary SMP-based microstructures, e.g., microchannels, microholes, and microwells, have been achieved with microfabrication. However, current SMP-based microstructures tend to be on the scale of hundreds of microns to millimeters. Considering the fact that SMPs possess the potential to create in situ controllable microscale actuators, hybrid SMP materials suitable for TPP should be developed.

#### 3.1.4. Material Processing

In addition to varying the type, material modification is also an important method for improving the performance of microactuators [129,130,131,132]. Ye studied the influence of the hydrophobicity of helical swimmers’ surfaces on their swimming performance through theoretical analysis and experiments. Researchers found that a helical swimmer with a more hydrophobic surface exerted less friction drag torque and exhibited a higher step-out frequency, indicating that the helical swimmer with a more hydrophobic surface should have had better swimming performance. Making a surface more hydrophobic may be a potential approach for improving the performance of 3D microactuators [133].

Given that an important application of microactuators is targeted drug delivery in vivo, reliable biocompatibility/biodegradation should be considered [13,106,134,135,136,137]. Traditionally, metal evaporation (e.g., Au) has been used to improve the affinity of microstructures to cells [107]. Furthermore, processed biological materials (e.g., dehydrated spirulina) [8,138,139] that are friendly to living cells/tissue have also been used as microrobots.

The material processing of printed 3D structures can also be achieved with chemical or physical methods. Oran developed metal nanoscaffolds via a controlled shrinkage method. A patterned and functionalized gel scaffold was shrunk by a factor of 10 to 20 in each dimension by using acid or divalent cations over a period of hours, and then it was dehydrated to achieve the desired nanoscale resolution [140]. Heating or sintering is also an effective processing strategy for removing excess materials with different melting points.

### 3.2. Structural Design and Fabrication

#### 3.2.1. Design and Optimization of Structural Parameters

The shape of its structure will affect the kinematic performance of a microactuator. Taking a spiral microswimmer as an example, inspired by the rotary motors of *E. coli* bacteria driving their bundles of flagella to rotate in a helical pattern and, in this way, generating propulsion, a helical shape has been considered as a classical structure for effective motion in fluid with a low Reynolds number (such as blood, stomach mucus, and tissue fluid) [141,142,143]. Currently, helical microswimmers adopt the same swimming mechanism as *E. coli* bacteria, substituting the internal rotary engine with an external magnetic field. The heights, diameters, taper angles, and pitch periods of a microhelix affect its swimming and loading capabilities [144]. Helix microactuators encounter the problem of lateral drift, which can be reduced by the optimization of the structural parameters. In the past few decades, researchers have designed and fabricated various types of helical microswimmers with different head shapes, magnetic positioning, and geometrical parameters for the heads and tails [145]. In addition, a number of studies have been established with respect to the influence of these variables on the swimming performance of helical microswimmers [133].

While the structures of other microactuators also affect their performance, the optimization of the structural parameters is a necessary process to improve the corresponding kinematic performance [38,146].

#### 3.2.2. Miniaturization of Actuators

Improving the machining accuracy is a direct way to promote the miniaturization of actuators [140]. Based on TPP, Gu’s group developed a three-dimensional optical beam lithography with a 9 nm feature size and 52 nm two-line resolution in a newly developed two-photon absorption resin with high mechanical strength [26]. For inorganic materials, Sun’s group achieved ultra-high resolution under atmospheric conditions without the detrimental ion doping that is inevitable in conventional FIB processing on dielectric and semiconductor materials via near-field optical methods [147]. A spatial resolution of less than 20 nm (λ/40, with λ being the light wavelength) is readily achieved, which opens a new avenue for the industrial production of nanopatterns and nanodevices using direct writing with ultrafast lasers.

It is worth noting that the resolution of the structure’s size can be enhanced by subsequent processing after 3D printing. Gong et al. proposed an implosion fabrication method to improve the resolution of printed hydrogel constructs through a complexation-induced construction without changing the printer hardware or most of the ink compositions [148]. Through the charge complexation and the subsequent expulsion of water from the gels, these printed constructs were found to have their linear dimensions reduced by various degrees. This method can also be applied in other 3D printing techniques, including direct extrusion printing, sacrificial printing, and microfluidic hollow fiber printing.

#### 3.2.3. Fabrication Method with Higher Production and Lower Cost

Although TPP is a precision technique used to fabricate 3D microstructures with complex shapes, its point-by-point scanning strategy leads to a low processing efficiency and practical potential [57,149,150]. Fortunately, effective fabrication can be achieved with the spatial light modulation (SLM) technique, which has resulted in a large amount of meaningful work [151,152,153,154,155]. For example, Li et al. presented a dynamic holographic processing method and splicing method to generate more complex pH-responsive microstructures (S-shaped and C-shaped microtubes, and torsional chiral structures) in about 10 s, which was faster than point-by-point manufacturing by 2–3 orders [86]. Xin et al. reported a precise and efficient method for the one-step synthesis of a 3D magnetic tubular micromotor. Three-dimensional biconical tubular micromotors were rapidly fabricated with a single exposure of ~100 ms with structured femtosecond optical vortices [72]. Compared with conventional tubular microstructure fabrication methods, this method, which allows for the 2D and 3D control of microtube geometry with sub-micrometer resolution, as well as high uniformity, features fast and high-throughput fabrication, while surpassing limitations such as complex processing procedures and contamination from processing chemicals.

In addition to a simple circular structure exposed by circular light field, more complex 3D structures can be realized with advanced light modulation strategies [156]. For example, Ni et al. proposed a coaxial interference technique of vortex and plane light, such that controlled 3D chiral microstructures in isotropic polymers were rapidly fabricated by one-step exposure [51]. By using this efficient single-exposure manufacturing technology, chiral microstructures with large area (>5 mm^2^) and high precision (~100 nm) were fabricated. Similarly, by superimposing vortex light phases with topological charge numbers in opposite directions, they also developed a method to divide the incident beam into an even number of two-dimensional focus arrays along a circular arc. In the aspect of controllable microcolumn processing, the bifurcation inclined microcolumn structures were fabricated by single exposure using this special light field, and a layered microcolumn array could be formed by controlling its spatial distribution [157]. This work provided a fast, stable, and easy-to-operate method for processing 3D microstructures in isotropic polymers.

With the development of optical field calculation methods, we believe that there will be more optical field exposure methods, which can replace point-by-point processing to greatly improve the machining efficiency of TPP for 3D microstructures [152].

In addition, stereolithography (SLA)-based 3D printing provides an alternative solution due to its mass-manufacturing capability. Compared with TPP, SLA-based 3D printing fabricates microstructures in a layer-by-layer fashion, which dramatically reduces the cost of time. By tuning the pattern of light and exposure dosage, the fabrication of microstructures with desired geometry and mechanical gradient can be achieved. Thus, SLA-based 3D printing exhibits a promising future for the mass manufacturing of functional microactuators and soft robots. However, the current resolution of SLA is not high enough to compete with TPP, which hinders further applications of SLA.

### 3.3. Prospective Actuation Methods for 3D Microactuators

The recent era has seen many impressive 3D microactuators fabricated using TPP and other methods. However, to date, most efforts are stuck at the laboratory stage and are a long way from practical applications. For turning these microactuators into realistic applications, more effective, wireless, and milder stimuli should be further developed [158]. Considering that one important application of outfield driven microactuators is drug delivery in medical field [159], we think that magnetic and ultrasound driving will be the focus of research [160,161,162]. Moreover, electric driving and the multi-field coupling method are promising future directions.

The future of magnetic driven microactuators, especially those involving biomedicine applications, may focus on three areas. First, the control objective can be changed from controlling a single or a small number of actuators to cluster control [163]. Mass administration requires a large number of microactuators to collaborate simultaneously [164]. Second, future applications can depend on the development of magnetic control system. The scaling issue, which includes not only the miniaturization of the electromagnetic control system, but also the accuracy of the magnetic control, should be addressed [165,166,167]. Third, an imaging system that can prepare feedback on the position and state of the microactuators is a must, and it should preferably be integrated into a miniaturized system, especially considering those tasks performed in vivo [159,168,169,170,171].

Correspondingly, acoustic waves are usually generated by piezoelectric transducers which are hard to reduce in size [172]. The 3D laser printing method may be a powerful tool with potential application in the fabrication of microgenerators that can transform electric force to mechanical force, resulting in an ultrasound field. Although ultrasound is a powerful, wireless, penetrating, and biosecurity tool which has been widely utilized in the medical domain [134], there are still some issues that need to be addressed for ultrasound-driven microactuators [173], for example, low precision. A main technical hurdle that needs to be overcome is unlocking the ability to manipulate particles < 100 nm in size. Another example is the inability to manipulate the *z*-axis. Although current methods may provide excellent control in planar translation, *z*-axis manipulation lacks the same dexterity and presents a future challenge. Furthermore, only collective manipulation is currently possible, whereas individual or selective manipulation from a swarm remains a challenge. Current acoustic-based methods lack the ability to selectively control individual cells or particles. Integrated research has only just begun and it depends on experience. In order to facilitate the increasing adoption of acoustic microfluidics by researchers, user-friendly, turnkey, integrated devices that perform routine laboratory processes with minimal professional training are necessary [105]. Although researchers have begun developing portable acoustic microfluidic tools that attempt to bridge the gap between development stages and ultimate use [174], no exciting advances have been reported so far. Lastly, current 3D microactuators printed by TPP lack the ability to convert ultrasound waves into apparent mechanical motion. More suitable materials, as well as corresponding technologies, are urgently required.

In view of a mature integrated circuit, electric-driven 3D microactuators may be employed more widely due to their advantages [52,175]. For example, the sensor signal needs to be changed into an electrical signal to facilitate processing, while electrically driven microactuators enable the actuator and sensor components to be integrated [52,176]. The two large gaps that need to be bridged for practical electrical-driven 3D microactuators are materials and manufacturing strategies [177,178,179]. It is difficult to construct 3D microcircuit and electric sensitive microactuators [180,181,182,183,184,185,186]. Currently, employing femtosecond laser writing, researchers have already built several constructive efforts. Qian et al. fabricated multi-pattern Ag microwires through femtosecond laser direct writing combined with multi-photon reduction technology, which can be used for microheaters [187]. Despite the microheater turning electricity into heat, converting electricity into the mechanical motion of the device is challenging. Potential ways to power electrically driven microactuators include a wireless power supply and integrated tiny batteries. However, the different processing methods for microcircuits and microactuators lead to serious material incompatibilities, whereby many high-performance battery materials cannot be easily processed on a chip [188]. In addition to “roll or fold” and “layer to layer” strategies for microelectrical appliances and tiny batteries, we consider mixing photosensitive materials with piezoelectric materials as one possible solution [180].

In order to adapt to more complex environments for practical application, microactuators that are responsive to dual stimuli and able to be controlled by multiple external powers were developed [44,189]. Correspondingly, this compound driving method can be named as multi-field-coupling manipulation. Currently, magnetic driving is the most popular driving method integrated with other methods due to its merits. For example, magnetic [190], moisture [189], light [191,192], and heat [6] driving mechanisms have been employed together as hybrid power sources of microactuators. Compared with strategies employing a single driving mechanism, multi-field-coupling manipulation presents obvious advantages so that the microactuators can perform more sophisticated tasks. For example, using magnetic driving to navigate a drug carrier and a specific acoustic signal to trigger the release of drug can achieve more precisely targeted slow release. Therefore, in the future, we will witness an explosion of multi-field-coupling methods for 3D microactuators.

## 4. Outlook

This review provided an overview of recent progress in TPP for functional microactuators. Representative stimulus methods and printable materials for TPP-based microactuators were introduced in detail. While considerable efforts have been made toward the practical applications of 3D printed microactuators, further advancements are still needed in the control and functionalization of microactuators. In this regard, several research opportunities can be taken into consideration. Firstly, the cooperative control of multi 3D printed microactuators will dramatically enhance their performance and promise benefits in performing coordinated tasks. The collective behavior of fish and the metachronal coordination of cilia array demonstrate the remarkable swarming intelligence of the biological world and provide fruitful inspiration for microactuator systems. A multi-agent actuator system that can be fabricated by high-throughput 3D printing techniques and advanced control algorithms are needed to achieve the emergent behaviors of the system. Secondly, further research and improvement of smart materials and 3D printing techniques are demanded to fabricate complex actuation systems with programmable and reconfigurable magnetic, acoustic, and mechanical properties such as a magnetic domain, acoustic impedance, and stiffness. The systems with tailored properties will enable them to adapt to different environments. Lastly, the integration of microactuators with an imaging modality will promote their practical application, especially for tasks in biological environments. The localization of microactuators in confined space is essential to execute real-time feedback control and ensure their safe operation. By doping imaging agents such as bubble or iron oxide nanoparticles during the 3D printing process, microactuators can be applied in clinical diagnostics.

In spite of the gaps to be filled, encouraging results are blooming with a growing number of enthusiastic researchers. It is believed that, with the rapid development of smart materials and advanced processing technologies, successive TPP fabrication will find broad applications in prototyping 3D microactuators employed in several fields.

## Figures and Tables

**Table 1:** Current mainstream 3D printing methods for 3D microstructures.

Method	Strategy	Resolution	Size	Material	Speed	Ref.
TPP	Direct writing	<500 nm	<mm	Single	Slow	[50]
	SLM	<500 nm	<mm	Single	Fast	[51]
Ink printing	Liquid ink	50 μm	>cm	Multi	Slow	[52]
Digital light processing	Point scanning	10 μm	cm	Single	Slow	[53]
Stereolithography	Layer	2 μm (*x*/*y*)	mm	Single	Medium	[54]
Liquid interface lithography	Layer	10 μm	cm	Single	Fast	[55]
Volumetric printing	Volumetric	50 μm	mm	Single	Very fast	[56]
Two-light printing	Layer	10 μm	cm	Single	fast	[57]

**Table 2:** Comparison of typical driving methods.

Driving Method	Control				Remarkable Superiority	Demerit
	Biological Penetration	Swarm	Separate from Swarm	Remote		
Magnetic	√	√	×	√	Excellent biological safety and penetration 3D collective manipulation	Complex and massive electromagnetic field equipment Cannot manipulate special target from a swarm
Light	Difficult	√	√	√	Enabling simultaneous and separate manipulation because of variable parameters (wavelength, intensity and phase et al.)	Poor biological penetration
pH	×	√	×	×	Simple and convenient operation	Most pH value is lethal to living cells or organisms
Microforce	×	Difficult	√	×	Universal for most materials	Must contact
Heat	Difficult	√	Difficult	√	Universal for most materials	Cannot manipulate special target from a swarm
Solution	×	√	×	×	Simple and convenient operation	Poor biocompatibility
Acoustic	√	√	Difficult	√	Excellent biological safety and penetration	Control precision *Z*-axis manipulation
Electric	×	√	√	√	Easy to integrate with mature IC	Functional materials and corresponding structures are difficult to miniaturize

**Table 3:** Summary of typical TPP-fabricated 3D microactuators.

Driving Method	Shape and Fabrication Method					Response Time	Application
	Shape	Feature Size	Fabrication				
		(μm)	Method	Material	Time		
Magnetic	Helix [41]	10 × 8.5 × 2	DLW	SU-8/IP-L	-	Real time	Delivery
	Helix [69]	16 × 5 × 2	DLW	IP-L	-		
	Helix [70]	10 × 5 × 1.5	DLW	IP-L	-		
	Screw [71]	70 × 40 × 5	DLW	TMPETA	-		
	Hollow helix [72]	75 × 15 × 5	FE-DLW	SZ2080	10 s		
	Tube [73]	80 × 25 × 1	FE-DLW	SZ2080	10 s		
Light	Gear [82]	20 × 20 × 2	DLW	-	-	-	Photonic
	Cantilever [83]	50 × 16 × 1.5	DLW	NIPAM-gel	-	0.03 s	-
	Gripper [84]	60 × 60 × 1.5	DLW	LCE	-	2 s	-
pH	Tuber [85]	50 × 10 × 3	FE-DLW	Hydrogel	10 s	<1 s	Microfluidic
	Tuber [86]	50 × 10 × 3	FE-DLW		10 s		Trapping
	Cantilever [24]	60 × 10 × 2	DLW		0.5 h		-
	Flower/Cage [87]	60 × 15 × 3	DLW		0.5 h		Trapping
Microforce	Pillar [91]	6 × 0.8 × 0.8	DLW	SZ2080	1 s	1 s	Trapping
	Gripper [93]	50 × 50 × 60	DLW	IP-Dip	-	Real time	Trapping
	Gear [94]	3 × 15 × 15	MF-DLW	SU-8	~0.5 h	10 s	-
Heat	Cantilever [96]	70 × 5 × 10	DLW	pNIPAM	-	-	-
	Flower [97]	6 × 70 × 70	DLW	CLC	-	-	Photonic
Solution	Rocket [102]	10 × 20 × 0.5	DLW	IP-L	-	-	-
	Flower [97]	6 × 70 × 70	DLW	CLC	-	~5 s	Photonic
Acoustic	Cantilever [109]	50 × 16 × 1.5	DLW	SU-8	-	Real time	Sensor
Electric	-		-			-	-

DLW: direct laser writing; MF-DLW: multi-foci direct laser writing; FE-DLW: field exposure + direct laser writing. LCE: liquid crystalline elastomer; CLC: supramolecular cholesteric liquid crystals.

## References

[1] Zhang Y., Li Y., Hu Y., Zhu X., Huang Y., Zhang Z., Rao S., Hu Z., Qiu W., Wang Y. (2018). Localized Self-Growth of Reconfigurable Architectures Induced by a Femtosecond Laser on a Shape-Memory Polymer. Adv. Mater..

[2] Gao W., Dong R., Thamphiwatana S., Li J., Gao W., Zhang L., Wang J. (2015). Artificial Micromotors in the Mouse’s Stomach: A Step toward in Vivo Use of Synthetic Motors. ACS Nano.

[3] Wang E., Desai M.S., Lee S.W. (2013). Light-controlled graphene-elastin composite hydrogel actuators. Nano Lett..

[4] Cui H., Zhao Q., Zhang L., Du X. (2020). Intelligent Polymer-Based Bioinspired Actuators: From Monofunction to Multifunction. Adv. Intell. Syst..

[5] Malachowski K., Jamal M., Jin Q., Polat B., Morris C.J., Gracias D.H. (2014). Self-folding single cell grippers. Nano Lett..

[6] Breger J.C., Yoon C., Xiao R., Kwag H.R., Wang M.O., Fisher J.P., Nguyen T.D., Gracias D.H. (2015). Self-folding thermo-magnetically responsive soft microgrippers. ACS Appl. Mater. Interfaces.

[7] Ceylan H., Yasa I.C., Yasa O., Tabak A.F., Giltinan J., Sitti M. (2019). 3D-Printed Biodegradable Microswimmer for Theranostic Cargo Delivery and Release. ACS Nano.

[8] Yan X., Xu J., Zhou Q., Jin D., Vong C.I., Feng Q., Ng D.H.L., Bian L., Zhang L. (2019). Molecular cargo delivery using multicellular magnetic microswimmers. Appl. Mater. Today.

[9] Jin D., Zhang L. (2020). Embodied intelligence weaves a better future. Nat. Mach. Intell..

[10] Hu N., Han X., Huang S., Grover H.M., Yu X., Zhang L.N., Trase I., Zhang J.X., Zhang L., Dong L.X. (2017). Edge effect of strained bilayer nanofilms for tunable multistability and actuation. Nanoscale.

[11] Nojoomi A., Arslan H., Lee K., Yum K. (2018). Bioinspired 3D structures with programmable morphologies and motions. Nat. Commun..

[12] Ma J.N., Zhang Y.L., Han D.D., Mao J.W., Chen Z.D., Sun H.B. (2020). Programmable deformation of patterned bimorph actuator swarm. Natl. Sci. Rev..

[13] Cangialosi A., Yoon C., Liu J., Huang Q., Guo J., Nguyen T.D., Gracias D.H., Schulman R. (2017). DNA sequence-directed shape change of photopatterned hydrogels via high-degree swelling. Science.

[14] Zolfagharian A., Kouzani A.Z., Khoo S.Y., Moghadam A.A.A., Gibson I., Kaynak A. (2016). Evolution of 3D printed soft actuators. Sens. Actuators A Phys..

[15] Highley C.B., Rodell C.B., Burdick J.A. (2015). Direct 3D Printing of Shear-Thinning Hydrogels into Self-Healing Hydrogels. Adv. Mater..

[16] Truby R.L., Lewis J.A. (2016). Printing soft matter in three dimensions. Nature.

[17] Schaffner M., Faber J.A., Pianegonda L., Ruhs P.A., Coulter F., Studart A.R. (2018). 3D printing of robotic soft actuators with programmable bioinspired architectures. Nat. Commun..

[18] Van Oosten C.L., Bastiaansen C.W., Broer D.J. (2009). Printed artificial cilia from liquid-crystal network actuators modularly driven by light. Nat. Mater..

[19] Ionov L. (2014). Hydrogel-based actuators: Possibilities and limitations. Mater. Today.

[20] Kim Y., Yuk H., Zhao R., Chester S.A., Zhao X. (2018). Printing ferromagnetic domains for untethered fast-transforming soft materials. Nature.

[21] Zhang S., Wang B., Jiang J., Wu K., Guo C.F., Wu Z. (2019). High-Fidelity Conformal Printing of 3D Liquid Alloy Circuits for Soft Electronics. ACS Appl. Mater. Interfaces.

[22] Ambulo C.P., Burroughs J.J., Boothby J.M., Kim H., Shankar M.R., Ware T.H. (2017). Four-dimensional Printing of Liquid Crystal Elastomers. ACS Appl. Mater. Interfaces.

[23] Gladman A.S., Matsumoto E.A., Nuzzo R.G., Mahadevan L., Lewis J.A. (2016). Biomimetic 4D printing. Nat. Mater..

[24] Jin D., Chen Q., Huang T.-Y., Huang J., Zhang L., Duan H. (2020). Four-dimensional direct laser writing of reconfigurable compound micromachines. Mater. Today.

[25] Kawata S., Sun H.-B., Tanaka T., Takada K. (2001). Finer features for functional microdevices. Nature.

[26] Gan Z., Cao Y., Evans R.A., Gu M. (2013). Three-dimensional deep sub-diffraction optical beam lithography with 9 nm feature size. Nat. Commun..

[27] Zhang Y.-L., Chen Q.-D., Xia H., Sun H.-B. (2010). Designable 3D nanofabrication by femtosecond laser direct writing. Nano Today.

[28] Zhang Y., Jiao Y., Li C., Chen C., Li J., Hu Y., Wu D., Chu J. (2020). Bioinspired micro/nanostructured surfaces prepared by femtosecond laser direct writing for multi-functional applications. Int. J. Extrem. Manuf..

[29] Zhang C., Hu Y., Li J., Lao Z., Ni J., Chu J., Huang W., Wu D. (2014). An improved multi-exposure approach for high quality holographic femtosecond laser patterning. Appl. Phys. Lett..

[30] Lao Z., Hu Y., Zhang C., Yang L., Li J., Chu J., Wu D. (2015). Capillary Force Driven Self-Assembly of Anisotropic Hierarchical Structures Prepared by Femtosecond Laser 3D Printing and Their Applications in Crystallizing Microparticles. ACS Nano.

[31] Xu B., Du W.Q., Li J.W., Hu Y.L., Yang L., Zhang C.C., Li G.Q., Lao Z.X., Ni J.C., Chu J.R. (2016). High efficiency integration of three-dimensional functional microdevices inside a microfluidic chip by using femtosecond laser multifoci parallel microfabrication. Sci. Rep..

[32] Zhang C., Hu Y., Du W., Wu P., Rao S., Cai Z., Lao Z., Xu B., Ni J., Li J. (2016). Optimized holographic femtosecond laser patterning method towards rapid integration of high-quality functional devices in microchannels. Sci. Rep..

[33] Lao Z.X., Hu Y.L., Pan D., Wang R.Y., Zhang C.C., Ni J.C., Xu B., Li J.W., Wu D., Chu J.R. (2017). Self-Sealed Bionic Long Microchannels with Thin Walls and Designable Nanoholes Prepared by Line-Contact Capillary-Force Assembly. Small.

[34] Xu B., Hu W., Du W., Hu Y., Zhang C., Lao Z., Ni J., Li J., Wu D., Chu J. (2017). Arch-like microsorters with multi-modal and clogging-improved filtering functions by using femtosecond laser multifocal parallel microfabrication. Opt. Express.

[35] Cai Z., Liu Y., Hu Y., Zhang C., Xu J., Ji S., Ni J., Lao Z., Li J., Zhao Y. (2018). Generation of colorful Airy beams and Airy imaging of letters via two-photon processed cubic phase plates. Opt. Lett..

[36] Hu Y., Zhang Y., Yuan H., Wang R., Jiang S., Lao Z., Li G., Wu D., Li J., Chu J. (2018). Capillary-assisted localized crystallization on discrete micropillar rings. Appl. Phys. Lett..

[37] Xu B., Shi Y., Lao Z., Ni J., Li G., Hu Y., Li J., Chu J., Wu D., Sugioka K. (2018). Real-Time Two-Photon Lithography in Controlled Flow to Create a Single-Microparticle Array and Particle-Cluster Array for Optofluidic Imaging. Lab Chip.

[38] Zhang Y.L., Tian Y., Wang H., Ma Z.C., Han D.D., Niu L.G., Chen Q.D., Sun H.B. (2019). Dual-3D Femtosecond Laser Nanofabrication Enables Dynamic Actuation. ACS Nano.

[39] Sun Y.L., Dong W.F., Yang R.Z., Meng X., Zhang L., Chen Q.D., Sun H.B. (2012). Dynamically tunable protein microlenses. Angew. Chem. Int. Ed..

[40] Liu D.-X., Sun Y.-L., Dong W.-F., Yang R.-Z., Chen Q.-D., Sun H.-B. (2014). Dynamic laser prototyping for biomimetic nanofabrication. Laser Photonics Rev..

[41] Tottori S., Zhang L., Qiu F., Krawczyk K.K., Franco-Obregon A., Nelson B.J. (2012). Magnetic helical micromachines: Fabrication, controlled swimming, and cargo transport. Adv. Mater..

[42] Yang Z., Zhang L. (2020). Magnetic Actuation Systems for Miniature Robots: A Review. Adv. Intell. Syst..

[43] Ionov L. (2015). Polymeric actuators. Langmuir.

[44] Wang Q., Zhang L. (2021). External Power-Driven Microrobotic Swarm: From Fundamental Understanding to Imaging-Guided Delivery. ACS Nano.

[45] Hu C., Pané S., Nelson B.J. (2018). Soft Micro- and Nanorobotics. Annu. Rev. Control Robot. Auton. Syst..

[46] Medina-Sánchez M., Magdanz V., Guix M., Fomin V.M., Schmidt O.G. (2018). Swimming Microrobots: Soft, Reconfigurable, and Smart. Adv. Funct. Mater..

[47] Zhang Y., Yuan K., Zhang L. (2019). Micro/Nanomachines: From Functionalization to Sensing and Removal. Adv. Mater. Technol..

[48] Sitti M., Wiersma D.S. (2020). Pros and Cons: Magnetic versus Optical Microrobots. Adv. Mater..

[49] Wang B., Kostarelos K., Nelson B.J., Zhang L. (2021). Trends in Micro-/Nanorobotics: Materials Development, Actuation, Localization, and System Integration for Biomedical Applications. Adv. Mater..

[50] Ma Z.C., Zhang Y.L., Han B., Hu X.Y., Li C.H., Chen Q.D., Sun H.B. (2020). Femtosecond laser programmed artificial musculoskeletal systems. Nat. Commun..

[51] Ni J., Wang C., Zhang C., Hu Y., Yang L., Lao Z., Xu B., Li J., Wu D., Chu J. (2017). Three-dimensional chiral microstructures fabricated by structured optical vortices in isotropic material. Light Sci. Appl..

[52] Zhao J., Lu H., Zhang Y., Yu S., Malyi O.I., Zhao X., Wang L., Wang H., Peng J., Li X. (2021). Direct coherent multi-ink printing of fabric supercapacitors. Sci. Adv..

[53] Ge Q., Chen Z., Cheng J., Zhang B., Zhang Y.-F., Li H., He X., Yuan C., Liu J., Magdassi S. (2021). 3D printing of highly stretchable hydrogel with diverse UV curable polymers. Sci. Adv..

[54] Yin H., Ding Y., Zhai Y., Tan W., Yin X. (2018). Orthogonal programming of heterogeneous micro-mechano-environments and geometries in three-dimensional bio-stereolithography. Nat. Commun..

[55] Tumbleston J.R., Shirvanyants D., Ermoshkin N., Janusziewicz R., Johnson A.R., Kelly D., Chen K., Pinschmidt R., Rolland J.P., Ermoshkin A. (2015). Continuous liquid interface production of 3D objects. Science.

[56] Shusteff M., Browar A.E.M., Kelly B.E., Henriksson J., Weisgraber T.H., Panas R.M., Fang N.X., Spadaccini C.M. (2017). One-step volumetric additive manufacturing of complex polymer structures. Sci. Adv..

[57] Regehly M., Garmshausen Y., Reuter M., Konig N.F., Israel E., Kelly D.P., Chou C.Y., Koch K., Asfari B., Hecht S. (2020). Xolography for linear volumetric 3D printing. Nature.

[58] Wang B., Chan K.F., Yu J., Wang Q., Yang L., Chiu P.W.Y., Zhang L. (2018). Reconfigurable Swarms of Ferromagnetic Colloids for Enhanced Local Hyperthermia. Adv. Funct. Mater..

[59] Zhou Q., Petit T., Choi H., Nelson B.J., Zhang L. (2017). Dumbbell Fluidic Tweezers for Dynamical Trapping and Selective Transport of Microobjects. Adv. Funct. Mater..

[60] Wang Q., Yang L., Wang B., Yu E., Yu J., Zhang L. (2019). Collective Behavior of Reconfigurable Magnetic Droplets via Dynamic Self-Assembly. ACS Appl. Mater. Interfaces.

[61] Ji F., Wang B., Zhang L. (2020). Light-Triggered Catalytic Performance Enhancement Using Magnetic Nanomotor Ensembles. Research.

[62] Wang Q., Yu J., Yuan K., Yang L., Jin D., Zhang L. (2020). Disassembly and spreading of magnetic nanoparticle clusters on uneven surfaces. Appl. Mater. Today.

[63] Harduf Y., Jin D., Or Y., Zhang L. (2018). Nonlinear Parametric Excitation Effect Induces Stability Transitions in Swimming Direction of Flexible Superparamagnetic Microswimmers. Soft Robot..

[64] Yu J., Xu T., Lu Z., Vong C.I., Zhang L. (2017). On-Demand Disassembly of Paramagnetic Nanoparticle Chains for Microrobotic Cargo Delivery. IEEE Trans. Robot..

[65] Yu J., Wang B., Du X., Wang Q., Zhang L. (2018). Ultra-extensible ribbon-like magnetic microswarm. Nat. Commun..

[66] Zhang Y., Yan K., Ji F., Zhang L. (2018). Enhanced Removal of Toxic Heavy Metals Using Swarming Biohybrid Adsorbents. Adv. Funct. Mater..

[67] Yu J., Jin D., Chan K.F., Wang Q., Yuan K., Zhang L. (2019). Active generation and magnetic actuation of microrobotic swarms in bio-fluids. Nat. Commun..

[68] Maruo S., Inoue H. (2006). Optically driven micropump produced by three-dimensional two-photon microfabrication. Appl. Phys. Lett..

[69] Qiu F., Fujita S., Mhanna R., Zhang L., Simona B.R., Nelson B.J. (2015). Magnetic Helical Microswimmers Functionalized with Lipoplexes for Targeted Gene Delivery. Adv. Funct. Mater..

[70] Medina-Sanchez M., Schwarz L., Meyer A.K., Hebenstreit F., Schmidt O.G. (2016). Cellular Cargo Delivery: Toward Assisted Fertilization by Sperm-Carrying Micromotors. Nano Lett..

[71] Yasa I.C., Tabak A.F., Yasa O., Ceylan H., Sitti M. (2019). 3D-Printed Microrobotic Transporters with Recapitulated Stem Cell Niche for Programmable and Active Cell Delivery. Adv. Funct. Mater..

[72] Xin C., Yang L., Li J., Hu Y., Qian D., Fan S., Hu K., Cai Z., Wu H., Wang D. (2019). Conical Hollow Microhelices with Superior Swimming Capabilities for Targeted Cargo Delivery. Adv. Mater..

[73] Yang L., Chen X., Wang L., Hu Z., Xin C., Hippler M., Zhu W., Hu Y., Li J., Wang Y. (2019). Targeted Single-Cell Therapeutics with Magnetic Tubular Micromotor by One-Step Exposure of Structured Femtosecond Optical Vortices. Adv. Funct. Mater..

[74] Han D.D., Zhang Y.L., Ma J.N., Liu Y.Q., Han B., Sun H.B. (2016). Light-Mediated Manufacture and Manipulation of Actuators. Adv. Mater..

[75] Martella D., Nocentini S., Nuzhdin D., Parmeggiani C., Wiersma D.S. (2017). Photonic Microhand with Autonomous Action. Adv. Mater..

[76] Wang W., Liu Y.-Q., Liu Y., Han B., Wang H., Han D.-D., Wang J.-N., Zhang Y.-L., Sun H.-B. (2017). Direct Laser Writing of Superhydrophobic PDMS Elastomers for Controllable Manipulation via Marangoni Effect. Adv. Funct. Mater..

[77] Tian Y., Zhang Y.L., Ku J.F., He Y., Xu B.B., Chen Q.D., Xia H., Sun H.B. (2010). High performance magnetically controllable microturbines. Lab Chip.

[78] Maggi C., Saglimbeni F., Dipalo M., De Angelis F., Di Leonardo R. (2015). Micromotors with asymmetric shape that efficiently convert light into work by thermocapillary effects. Nat. Commun..

[79] Zeng H., Wasylczyk P., Wiersma D.S., Priimagi A. (2018). Light Robots: Bridging the Gap between Microrobotics and Photomechanics in Soft Materials. Adv. Mater..

[80] Pan D., Wu D., Li P.J., Ji S.Y., Nie X., Fan S.Y., Chen G.Y., Zhang C.C., Xin C., Xu B. (2021). Transparent Light-Driven Hydrogel Actuator Based on Photothermal Marangoni Effect and Buoyancy Flow for Three-Dimensional Motion. Adv. Funct. Mater..

[81] Han B., Zhang Y.-L., Chen Q.-D., Sun H.-B. (2018). Carbon-Based Photothermal Actuators. Adv. Funct. Mater..

[82] Lin X.F., Hu G.Q., Chen Q.D., Niu L.G., Li Q.S., Ostendorf A., Sun H.B. (2012). A light-driven turbine-like micro-rotor and study on its light-to-mechanical power conversion efficiency. Appl. Phys. Lett..

[83] Zheng C., Jin F., Zhao Y., Zheng M., Liu J., Dong X., Xiong Z., Xia Y., Duan X. (2020). Light-driven micron-scale 3D hydrogel actuator produced by two-photon polymerization microfabrication. Sens. Actuators B Chem..

[84] Chen L., Dong Y., Tang C.Y., Zhong L., Law W.C., Tsui G.C.P., Yang Y., Xie X. (2019). Development of Direct-Laser-Printable Light-Powered Nanocomposites. ACS Appl. Mater. Interfaces.

[85] Hu K., Yang L., Jin D., Li J., Ji S., Xin C., Hu Y., Wu D., Zhang L., Chu J. (2019). Tunable microfluidic device fabricated by femtosecond structured light for particle and cell manipulation. Lab Chip.

[86] Li R., Jin D., Pan D., Ji S., Xin C., Liu G., Fan S., Wu H., Li J., Hu Y. (2020). Stimuli-Responsive Actuator Fabricated by Dynamic Asymmetric Femtosecond Bessel Beam for In Situ Particle and Cell Manipulation. ACS Nano.

[87] Hu Y., Wang Z., Jin D., Zhang C., Sun R., Li Z., Hu K., Ni J., Cai Z., Pan D. (2020). Botanical-Inspired 4D Printing of Hydrogel at the Microscale. Adv. Funct. Mater..

[88] Hu Y.L., Yuan H.W., Liu S.L., Ni J.C., Lao Z.X., Xin C., Pan D., Zhang Y.Y., Zhu W.L., Li J.W. (2020). Chiral Assemblies of Laser-Printed Micropillars Directed by Asymmetrical Capillary Force. Adv. Mater..

[89] Lao Z., Zheng Y., Dai Y., Hu Y., Ni J., Ji S., Cai Z., Smith Z.J., Li J., Zhang L. (2020). Nanogap Plasmonic Structures Fabricated by Switchable Capillary-Force Driven Self-Assembly for Localized Sensing of Anticancer Medicines with Microfluidic SERS. Adv. Funct. Mater..

[90] Liu X.J., Gu H.C., Ding H.B., Du X., Wei M.X., Chen Q., Gu Z.Z. (2020). 3D Bioinspired Microstructures for Switchable Repellency in both Air and Liquid. Adv. Sci..

[91] Hu Y., Lao Z., Cumming B.P., Wu D., Li J., Liang H., Chu J., Huang W., Gu M. (2015). Laser printing hierarchical structures with the aid of controlled capillary-driven self-assembly. Proc. Natl. Acad. Sci. USA.

[92] Lao Z., Pan D., Yuan H., Ni J., Ji S., Zhu W., Hu Y., Li J., Wu D., Chu J. (2018). Mechanical-Tunable Capillary-Force-Driven Self-Assembled Hierarchical Structures on Soft Substrate. ACS Nano.

[93] Power M., Thompson A.J., Anastasova S., Yang G.Z. (2018). A Monolithic Force-Sensitive 3D Microgripper Fabricated on the Tip of an Optical Fiber Using 2-Photon Polymerization. Small.

[94] Vizsnyiczai G., Frangipane G., Maggi C., Saglimbeni F., Bianchi S., Di Leonardo R. (2017). Light controlled 3D micromotors powered by bacteria. Nat. Commun..

[95] Jia H., Mailand E., Zhou J., Huang Z., Dietler G., Kolinski J.M., Wang X., Sakar M.S. (2019). Universal Soft Robotic Microgripper. Small.

[96] Hippler M., Blasco E., Qu J., Tanaka M., Barner-Kowollik C., Wegener M., Bastmeyer M. (2019). Controlling the shape of 3D microstructures by temperature and light. Nat. Commun..

[97] Del Pozo M., Delaney C., Bastiaansen C.W.M., Diamond D., Schenning A., Florea L. (2020). Direct Laser Writing of Four-Dimensional Structural Color Microactuators Using a Photonic Photoresist. ACS Nano.

[98] Kagan D., Balasubramanian S., Wang J. (2011). Chemically triggered swarming of gold microparticles. Angew. Chem. Int. Ed..

[99] Xu T., Soto F., Gao W., Dong R., Garcia-Gradilla V., Magana E., Zhang X., Wang J. (2015). Reversible swarming and separation of self-propelled chemically powered nanomotors under acoustic fields. J. Am. Chem. Soc..

[100] Wang B., Ji F., Yu J., Yang L., Wang Q., Zhang L. (2019). Bubble-Assisted Three-Dimensional Ensemble of Nanomotors for Improved Catalytic Performance. iScience.

[101] Baker R.D., Montenegro-Johnson T., Sediako A.D., Thomson M.J., Sen A., Lauga E., Aranson I.S. (2019). Shape-programmed 3D printed swimming microtori for the transport of passive and active agents. Nat. Commun..

[102] Chen Y., Xu B., Mei Y. (2019). Design and Fabrication of Tubular Micro/Nanomotors via 3D Laser Lithography. Chem. Asian J..

[103] Belling J.N., Heidenreich L.K., Tian Z., Mendoza A.M., Chiou T.T., Gong Y., Chen N.Y., Young T.D., Wattanatorn N., Park J.H. (2020). Acoustofluidic sonoporation for gene delivery to human hematopoietic stem and progenitor cells. Proc. Natl. Acad. Sci. USA.

[104] Ozcelik A., Rufo J., Guo F., Gu Y., Li P., Lata J., Huang T.J. (2018). Acoustic tweezers for the life sciences. Nat. Methods.

[105] Zhang P., Bachman H., Ozcelik A., Huang T.J. (2020). Acoustic Microfluidics. Annu. Rev. Anal. Chem..

[106] Aliabouzar M., Zhang L.G., Sarkar K. (2016). Lipid Coated Microbubbles and Low Intensity Pulsed Ultrasound Enhance Chondrogenesis of Human Mesenchymal Stem Cells in 3D Printed Scaffolds. Sci. Rep..

[107] De Avila B.E.F., Angsantikul P., Ramirez-Herrera D.E., Soto F., Teymourian H., Dehaini D., Chen Y.J., Zhang L.F., Wang J. (2018). Hybrid biomembrane-functionalized nanorobots for concurrent removal of pathogenic bacteria and toxins. Sci. Robot..

[108] Wang Q., Zhang L. (2020). Ultrasound Imaging and Tracking of Micro/Nanorobots: From Individual to Collectives. IEEE Open J. Nanotechnol..

[109] Li M., Liu Y., Zhao X., Gao R., Li Y., Qu S. (2017). High sensitivity fiber acoustic sensor tip working at 1550 nm fabricated by two-photon polymerization technique. Sens. Actuators A Phys..

[110] Barik A., Zhang Y., Grassi R., Nadappuram B.P., Edel J.B., Low T., Koester S.J., Oh S.H. (2017). Graphene-edge dielectrophoretic tweezers for trapping of biomolecules. Nat. Commun..

[111] Mao G., Drack M., Karami-Mosammam M., Wirthl D., Stockinger T., Schwödiauer R., Kaltenbrunner M. (2020). Soft electromagnetic actuators. Sci. Adv..

[112] Chortos A., Hajiesmaili E., Morales J., Clarke D.R., Lewis J.A. (2019). 3D Printing of Interdigitated Dielectric Elastomer Actuators. Adv. Funct. Mater..

[113] Morales D., Palleau E., Dickey M.D., Velev O.D. (2014). Electro-actuated hydrogel walkers with dual responsive legs. Soft Matter.

[114] Yang C., Wang W., Yao C., Xie R., Ju X.J., Liu Z., Chu L.Y. (2015). Hydrogel Walkers with Electro-Driven Motility for Cargo Transport. Sci. Rep..

[115] Choi M.Y., Shin Y., Lee H.S., Kim S.Y., Na J.H. (2020). Multipolar spatial electric field modulation for freeform electroactive hydrogel actuation. Sci. Rep..

[116] Lao Z., Sun R., Jin D., Ren Z., Xin C., Zhang Y., Jiang S., Zhang Y., Zhang L. (2021). Encryption/decryption and microtarget capturing by pH driven Janus microstructures fabricated by same femtosecond laser printing parameters. Int. J. Extrem. Manuf..

[117] Carlotti M., Mattoli V. (2019). Functional Materials for Two-Photon Polymerization in Microfabrication. Small.

[118] Manouras T., Vamvakaki M. (2017). Field responsive materials: Photo-, electro-, magnetic- and ultrasound-sensitive polymers. Polym. Chem..

[119] Wang T., Torres D., Fernández F.E., Wang C., Sepúlveda N. (2017). Maximizing the performance of photothermal actuators by combining smart materials with supplementary advantages. Sci. Adv..

[120] Yeung K.-W., Dong Y., Chen L., Tang C.-Y., Law W.-C., Tsui G.C.-P., Engstrøm D.S. (2020). Printability of photo-sensitive nanocomposites using two-photon polymerization. Nanotechnol. Rev..

[121] Yang L., Zhang L. (2020). Motion Control in Magnetic Microrobotics: From Individual and Multiple Robots to Swarms. Annu. Rev. Control Robot. Auton. Syst..

[122] Wang Q., Yang L., Yu J., Chiu P.W.Y., Zheng Y.P., Zhang L. (2020). Real-Time Magnetic Navigation of a Rotating Colloidal Microswarm Under Ultrasound Guidance. IEEE Trans. Biomed. Eng..

[123] Yang L., Yu J., Zhang L. (2020). Statistics-Based Automated Control for a Swarm of Paramagnetic Nanoparticles in 2-D Space. IEEE Trans. Robot..

[124] Yang L., Zhang Y., Wang Q., Chan K.-F., Zhang L. (2020). Automated Control of Magnetic Spore-Based Microrobot Using Fluorescence Imaging for Targeted Delivery With Cellular Resolution. IEEE Trans. Autom. Sci. Eng..

[125] Alcantara C.C.J., Landers F.C., Kim S., De Marco C., Ahmed D., Nelson B.J., Pane S. (2020). Mechanically interlocked 3D multi-material micromachines. Nat. Commun..

[126] Zhang C., Lu X., Fei G., Wang Z., Xia H., Zhao Y. (2019). 4D Printing of a Liquid Crystal Elastomer with a Controllable Orientation Gradient. ACS Appl. Mater. Interfaces.

[127] Chen P., Wei B.Y., Hu W., Lu Y.Q. (2019). Liquid-Crystal-Mediated Geometric Phase: From Transmissive to Broadband Reflective Planar Optics. Adv. Mater..

[128] Yang H., Leow W.R., Wang T., Wang J., Yu J., He K., Qi D., Wan C., Chen X. (2017). 3D Printed Photoresponsive Devices Based on Shape Memory Composites. Adv. Mater..

[129] Wang X., Hu C., Schurz L., De Marco C., Chen X., Pane S., Nelson B.J. (2018). Surface-Chemistry-Mediated Control of Individual Magnetic Helical Microswimmers in a Swarm. ACS Nano.

[130] Kim E., Yoo S.J., Kim E., Kwon T.H., Zhang L., Moon C., Choi H. (2016). Nano-patterned SU-8 surface using nanosphere-lithography for enhanced neuronal cell growth. Nanotechnology.

[131] Wang B., Zhang Y., Zhang L. (2017). Selective surface tension induced patterning on flexible textiles via click chemistry. Nanoscale.

[132] Kang H., Jung H.J., Wong D.S.H., Kim S.K., Lin S., Chan K.F., Zhang L., Li G., Dravid V.P., Bian L. (2018). Remote Control of Heterodimeric Magnetic Nanoswitch Regulates the Adhesion and Differentiation of Stem Cells. J. Am. Chem. Soc..

[133] Ye C., Liu J., Wu X., Wang B., Zhang L., Zheng Y., Xu T. (2019). Hydrophobicity Influence on Swimming Performance of Magnetically Driven Miniature Helical Swimmers. Micromachines.

[134] Wang D., Gao C., Zhou C., Lin Z., He Q. (2020). Leukocyte Membrane-Coated Liquid Metal Nanoswimmers for Actively Targeted Delivery and Synergistic Chemophotothermal Therapy. Research.

[135] Alapan Y., Yasa O., Schauer O., Giltinan J., Tabak A.F., Sourjik V., Sitti M. (2018). Soft erythrocyte-based bacterial microswimmers for cargo delivery. Sci. Robot..

[136] Cabanach P., Pena-Francesch A., Sheehan D., Bozuyuk U., Yasa O., Borros S., Sitti M. (2020). Zwitterionic 3D-Printed Non-Immunogenic Stealth Microrobots. Adv. Mater..

[137] Wong D.S., Li J., Yan X., Wang B., Li R., Zhang L., Bian L. (2017). Magnetically Tuning Tether Mobility of Integrin Ligand Regulates Adhesion, Spreading, and Differentiation of Stem Cells. Nano Lett..

[138] Yan X., Zhou Q., Yu J., Xu T., Deng Y., Tang T., Feng Q., Bian L., Zhang Y., Ferreira A. (2015). Magnetite Nanostructured Porous Hollow Helical Microswimmers for Targeted Delivery. Adv. Funct. Mater..

[139] Yan X., Zhou Q., Vincent M., Deng Y., Yu J., Xu J., Xu T., Tang T., Bian L., Wang Y.J. (2017). Multifunctional biohybrid magnetite microrobots for imaging-guided therapy. Sci. Robot..

[140] Oran D., Rodriques S.G., Gao R., Asano S., Skylar-Scott M.A., Chen F., Tillberg P.W., Marblestone A.H., Boyden E.S. (2018). 3D nanofabrication by volumetric deposition and controlled shrinkage of patterned scaffolds. Science.

[141] Dai L., Huang X., Zhang L., Zhang L., Ge L. (2015). Mechanical properties of normal and binormal double nanohelices. RSC Adv..

[142] Liu L., Zhang L., Kim S.M., Park S. (2014). Helical metallic micro- and nanostructures: Fabrication and application. Nanoscale.

[143] Dai L., Zhu K.D., Shen W., Huang X., Zhang L., Goriely A. (2018). Controllable rotational inversion in nanostructures with dual chirality. Nanoscale.

[144] Dong Y., Wang L., Wang J., Wang S., Wang Y., Jin D., Chen P., Du W., Zhang L., Liu B.F. (2020). Graphene-Based Helical Micromotors Constructed by “Microscale Liquid Rope-Coil Effect” with Microfluidics. ACS Nano.

[145] Xu T., Yu J., Vong C.-I., Wang B., Wu X., Zhang L. (2019). Dynamic Morphology and Swimming Properties of Rotating Miniature Swimmers With Soft Tails. IEEE ASME Trans. Mechatron..

[146] Huang T.-Y., Huang H.-W., Jin D.D., Chen Q.Y., Huang J.Y., Zhang L., Duan H.L. (2020). Four-dimensional micro-building blocks. Sci. Adv..

[147] Li Z.Z., Wang L., Fan H., Yu Y.H., Sun H.B., Juodkazis S., Chen Q.D. (2020). O-FIB: Far-field-induced near-field breakdown for direct nanowriting in an atmospheric environment. Light Sci. Appl..

[148] Gong J., Schuurmans C.C.L., Genderen A.M.V., Cao X., Li W., Cheng F., He J.J., Lopez A., Huerta V., Manriquez J. (2020). Complexation-induced resolution enhancement of 3D-printed hydrogel constructs. Nat. Commun..

[149] Ge Q., Li Z.Q., Wang Z.L., Kowsari K., Zhang W., He X., Zhou J., Fang X. (2020). Projection micro stereolithography based 3D printing and its applications. Int. J. Extrem. Manuf..

[150] Traugutt N.A., Mistry D., Luo C., Yu K., Ge Q., Yakacki C.M. (2020). Liquid-Crystal-Elastomer-Based Dissipative Structures by Digital Light Processing 3D Printing. Adv. Mater..

[151] Cai Z., Qi X., Pan D., Ji S., Ni J., Lao Z., Xin C., Li J., Hu Y., Wu D. (2020). Dynamic Airy imaging through high-efficiency broadband phase microelements by femtosecond laser direct writing. Photonics Res..

[152] Hu Y., Wang Z., Wang X., Ji S., Zhang C., Li J., Zhu W., Wu D., Chu J. (2020). Efficient full-path optical calculation of scalar and vector diffraction using the Bluestein method. Light Sci. Appl..

[153] Pan D., Xu B., Liu S., Li J., Hu Y., Wu D., Chu J. (2020). Amplitude-phase optimized long depth of focus femtosecond axilens beam for single-exposure fabrication of high-aspect-ratio microstructures. Opt. Lett..

[154] Hu Y., Feng W., Xue C., Lao Z., Ji S., Cai Z., Zhu W., Li J., Wu D., Chu J. (2020). Self-assembled micropillars fabricated by holographic femtosecond multi-foci beams forin situ trapping of microparticles. Opt. Lett..

[155] Saha S.K., Wang D., Nguyen V.H., Chang Y., Oakdale J.S., Chen S.C. (2019). Scalable submicrometer additive manufacturing. Science.

[156] Wang C., Yang L., Hu Y., Rao S., Wang Y., Pan D., Ji S., Zhang C., Su Y., Zhu W. (2019). Femtosecond Mathieu Beams for Rapid Controllable Fabrication of Complex Microcages and Application in Trapping Microobjects. ACS Nano.

[157] Ni J.C., Wang Z.Y., Li Z.Q., Lao Z.X., Hu Y.L., Ji S.Y., Xu B., Zhang C.C., Li J.W., Wu D. (2017). Multifurcate Assembly of Slanted Micropillars Fabricated by Superposition of Optical Vortices and Application in High-Efficiency Trapping Microparticles. Adv. Funct. Mater..

[158] Hines L., Petersen K., Lum G.Z., Sitti M. (2017). Soft Actuators for Small-Scale Robotics. Adv. Mater..

[159] Wang B., Chan K.F., Yuan K., Wang Q., Xia X., Yang L., Ko H., Wang Y.-X.J., Sung J.J.Y., Chiu P.W.Y. (2021). Endoscopy-assisted magnetic navigation of biohybrid soft microrobots with rapid endoluminal delivery and imaging. Sci. Robot..

[160] Ji F., Li T., Yu S., Wu Z., Zhang L. (2021). Propulsion Gait Analysis and Fluidic Trapping of Swinging Flexible Nanomotors. ACS Nano.

[161] Zhou H., Mayorga-Martinez C.C., Pane S., Zhang L., Pumera M. (2021). Magnetically Driven Micro and Nanorobots. Chem. Rev..

[162] Dong Y., Wang L., Yuan K., Ji F., Gao J., Zhang Z., Du X., Tian Y., Wang Q., Zhang L. (2021). Magnetic Microswarm Composed of Porous Nanocatalysts for Targeted Elimination of Biofilm Occlusion. ACS Nano.

[163] Du X., Yu J., Jin D., Chiu P.W.Y., Zhang L. (2021). Independent Pattern Formation of Nanorod and Nanoparticle Swarms under an Oscillating Field. ACS Nano.

[164] Jin D., Yu J., Yuan K., Zhang L. (2019). Mimicking the Structure and Function of Ant Bridges in a Reconfigurable Microswarm for Electronic Applications. ACS Nano.

[165] Yang L., Wang Q., Vong C.-I., Zhang L. (2017). A Miniature Flexible-Link Magnetic Swimming Robot with Two Vibration Modes: Design, Modeling and Characterization. IEEE Robot. Autom. Let..

[166] Du X., Zhang M., Yu J., Yang L., Chiu W.Y.P., Zhang L. (2020). Design and Real-time Optimization for a Magnetic Actuation System with Enhanced Flexibility. IEEE-ASME Trans. Mechatron..

[167] Wu X., Liu J., Huang C., Su M., Xu T. (2020). 3-D Path Following of Helical Microswimmers With an Adaptive Orientation Compensation Model. IEEE Trans. Autom. Sci. Eng..

[168] Zhang Y., Zhang L., Yang L., Vong C.I., Chan K.F., Wu W.K.K., Kwong T.N.Y., Lo N.W.S., Ip M., Wong S.H. (2019). Real-time tracking of fluorescent magnetic spore-based microrobots for remote detection of C. diff toxins. Sci. Adv..

[169] Yang Z., Yang L., Zhang L. (2020). 3-D Visual Servoing of Magnetic Miniature Swimmers Using Parallel Mobile Coils. IEEE Trans. Med. Robot. Bionics.

[170] Wang Q., Chan K.F., Schweizer K., Du X., Jin D., Yu S.C.H., Nelson B.J., Zhang L. (2021). Ultrasound Doppler-guided real-time navigation of a magnetic microswarm for active endovascular delivery. Sci. Adv..

[171] Xu T., Guan Y., Liu J., Wu X. (2020). Image-Based Visual Servoing of Helical Microswimmers for Planar Path Following. IEEE Trans. Autom. Sci. Eng..

[172] Greenhall J., Raeymaekers B. (2017). 3D Printing Macroscale Engineered Materials Using Ultrasound Directed Self-Assembly and Stereolithography. Adv. Mater. Technol..

[173] Ding X., Lin S.C., Kiraly B., Yue H., Li S., Chiang I.K., Shi J., Benkovic S.J., Huang T.J. (2012). On-chip manipulation of single microparticles, cells, and organisms using surface acoustic waves. Proc. Natl. Acad. Sci. USA.

[174] Gu Y., Chen C., Rufo J., Shen C., Wang Z., Huang P.H., Fu H., Zhang P., Cummer S.A., Tian Z. (2020). Acoustofluidic Holography for Micro- to Nanoscale Particle Manipulation. ACS Nano.

[175] Ma Y., Sikdar D., Fedosyuk A., Velleman L., Klemme D.J., Oh S.H., Kucernak A.R.J., Kornyshev A.A., Edel J.B. (2020). Electrotunable Nanoplasmonics for Amplified Surface Enhanced Raman Spectroscopy. ACS Nano.

[176] Alblalaihid K., Overton J., Lawes S., Kinnell P. (2017). A 3D-printed polymer micro-gripper with self-defined electrical tracks and thermal actuator. J. Micromech. Microeng..

[177] Cheng J., Chen Y., Wu J.W., Ji X.R., Wu S.H. (2019). 3D Printing of BaTiO3 Piezoelectric Ceramics for a Focused Ultrasonic Array. Sensors.

[178] Park Y.-G., An H.S., Kim J.-Y., Park J.-U. (2019). High-resolution, reconfigurable printing of liquid metals with three-dimensional structures. Sci. Adv..

[179] Todaro C.J., Easton M.A., Qiu D., Zhang D., Bermingham M.J., Lui E.W., Brandt M., StJohn D.H., Qian M. (2020). Grain structure control during metal 3D printing by high-intensity ultrasound. Nat. Commun..

[180] Fogel O., Winter S., Benjamin E., Krylov S., Kotler Z., Zalevsky Z. (2018). 3D printing of functional metallic microstructures and its implementation in electrothermal actuators. Add. Manuf..

[181] Valentine A.D., Busbee T.A., Boley J.W., Raney J.R., Chortos A., Kotikian A., Berrigan J.D., Durstock M.F., Lewis J.A. (2017). Hybrid 3D Printing of Soft Electronics. Adv. Mater..

[182] Zhang L., Golod S.V., Deckardt E., Prinz V., Grützmacher D. (2004). Free-standing Si/SiGe micro- and nano-objects. Phys. E.

[183] Zhang L., Dong L., Bell D.J., Nelson B.J., Schönenberger C., Grützmacher D. (2006). Fabrication and characterization of freestanding Si/Cr micro-and nanospirals. Microelectron. Eng..

[184] Zhang L., Deckhardt E., Weber A., Schönenberger C., Grützmacher D. (2005). Controllable fabrication of SiGe/Si and SiGe/Si/Cr helical nanobelts. Nanotechnology.

[185] Zhang L., Ruh E., Grützmacher D., Dong L., Bell D.J., Nelson B.J., Schönenberger C. (2006). Anomalous coiling of sige/si and sige/si/cr helical nanobelts. Nano Lett..

[186] Bell D.J., Sun Y., Zhang L., Dong L.X., Nelson B.J., Grützmacher D. (2006). Three-dimensional nanosprings for electromechanical sensors. Sens. Actuators A Phys..

[187] Qian D., Yang L., Zhang Y., Xin C., Hu Z., Hu K., Wang Y., Pan D., Li J., Wu D. (2018). Flexible and rapid fabrication of silver microheaters with spatial-modulated multifoci by femtosecond laser multiphoton reduction. Opt. Lett..

[188] Zhu M., Schmidt O.G. (2021). Tiny robots and sensors need tiny batteries—Here’s how to do it. Nature.

[189] Gao Y.Y., Zhang Y.L., Han B., Zhu L., Dong B., Sun H.B. (2019). Gradient Assembly of Polymer Nanospheres and Graphene Oxide Sheets for Dual-Responsive Soft Actuators. ACS Appl. Mater. Interfaces.

[190] Lu H., Zhang M., Yang Y., Huang Q., Fukuda T., Wang Z., Shen Y. (2018). A bioinspired multilegged soft millirobot that functions in both dry and wet conditions. Nat. Commun..

[191] Nocentini S., Parmeggiani C., Martella D., Wiersma D.S. (2018). Optically Driven Soft Micro Robotics. Adv. Opt. Mater..

[192] Ji F., Jin D., Wang B., Zhang L. (2020). Light-Driven Hovering of a Magnetic Microswarm in Fluid. ACS Nano.

